# Simultaneous Recognition and Separation of Organic Isomers Via Cooperative Control of Pore‐Inside and Pore‐Outside Interactions

**DOI:** 10.1002/advs.202204963

**Published:** 2022-10-28

**Authors:** Shaomin Xue, Yujia Rong, Ning Ding, Chaofeng Zhao, Qi Sun, Shenghua Li, Siping Pang

**Affiliations:** ^1^ School of Materials Science and Engineering Beijing Institute of Technology Beijing 100081 P. R. China; ^2^ Yangtze Delta Region Academy Beijing Institute of Technology Jiaxing 314019 P. R. China

**Keywords:** intermolecular interactions, organic isomer, pillararenes, recognition, separation

## Abstract

Despite the desirability of organic isomer recognition and separation, current strategies are expensive and complicated. Here, a simple strategy for simultaneously recognizing and separating organic isomers using pillararene‐based charge‐transfer cocrystals through the cooperative control of pore‐inside and pore‐outside intermolecular interactions is presented. This strategy is illustrated using 1‐bromobutane (1‐BBU), which is often produced as an isomeric mixture with 2‐bromobutane (2‐BBU). According to its structure, perethylated pillar[5]arene (EtP5) and 3,5‐dinitrobenzonitrile (DNB) are strategically chosen as a donor and an acceptor. As a result, their cocrystal exhibited stronger pore‐inside interactions and much weaker pore‐outside interactions with 1‐BBU than with 2‐BBU. Consequently, nearly 100% 1‐BBU selectivity is achieved in two‐component mixtures, even in those containing trace 1‐BBU (1%), whereas free EtP5 only achieved 89.80% selectivity. The preference for linear bromoalkanes is retained in 1‐bromopentane/3‐bromopentane and 1‐bromohexane/2‐bromohexane mixtures, demonstrating the generality of this strategy. Selective adsorption of linear bromoalkanes induced a naked‐eye‐detectable color change from red to white. Moreover, the cocrystal are used over multiple cycles without losing selectivity.

## Graphical abstract

Perethylated pillar[5]arene (EtP5) and 3,5‐dinitrobenzonitrile are strategically chosen as a donor and an acceptor to form charge‐transfer cocrystals for simultaneous separation and recognition of bromoalkane isomers through cooperative control of pore‐inside and pore‐outside interactions.

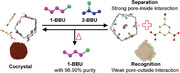

## 1 Introduction

**Figure 1:**
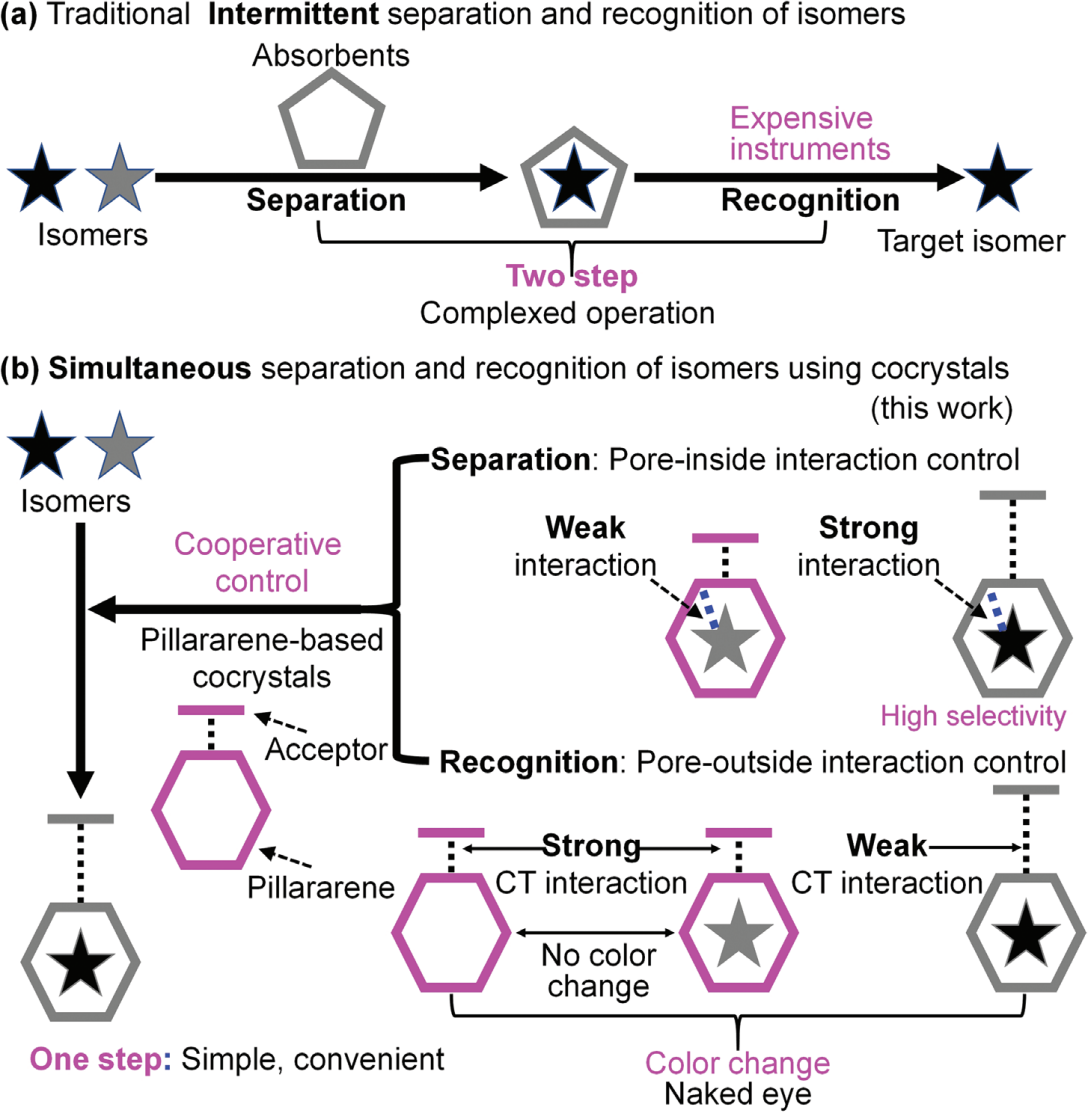
Strategies for recognition and separation of organic isomers. a) Traditional complex and expensive two‐step strategy. b) Simple and convenient one‐step strategy using pillararene‐based cocrystals through cooperative control of pore‐inside and pore‐outside intermolecular interactions (this work).

The separation of small organic isomers, including xylenes,^[^ ^1^ ^]^ alkanes,^[^ ^2^ ^]^ and haloalkanes,^[^ ^3^ ^]^ is a critical step in industrial processes because almost every isomer is a high‐value chemical feedstock in synthetic chemistry and the petrochemical industry. However, these isomers are usually produced from the same starting materials and obtained as mixtures. Owing to their identical molecular weights and very similar structures and properties, separation to obtain a single isomer with high purity remains exceedingly difficult.^[^ ^4^ ^]^ Commonly, such mixtures are separated using adsorption methods, which consume low amounts of energy and are environmentally friendly. Numerous adsorbents, including metal–organic frameworks,^[^ ^5^ ^]^ covalent organic frameworks,^[^ ^6^ ^]^ MFI zeolite membranes,^[^ ^7^ ^]^ and organic cages,^[^ ^8^ ^]^ have been developed and investigated for the separation of small organic isomers, such as p‐xylene/o‐xylene,^[^ ^9^ ^]^ 2‐methylpentane/3‐methylpentane,^[^ ^10^ ^]^ 1‐bromopropane/2‐bromopropane,^[^ ^11^ ^]^ and 1‐butane/2‐butane.^[^ ^2c^ ^]^ Despite exhibiting remarkable separation performance, most of these adsorbents lack the ability to recognize the entrapped isomer. After separation, recognition often requires the use of expensive instrumental methods, including gas‐phase mass spectrometry and nuclear magnetic resonance (NMR) spectroscopy, and complicated operations (e.g., multistep sample treatment; **Figure** **1**a).^[^ ^12^ ^]^ Moreover, because of their extensive applications and high volatility, these organic isomers are often found in the atmosphere, particularly in production workshops, which not only leads to significant environmental problems, such as photochemical smog and ozonosphere holes, but also causes direct and indirect harm to the human body.^[^ ^13^ ^]^ In this context, we envisioned that the development of new functional materials that simultaneously recognize and separate organic isomers would be highly desirable for fundamental research and industrial production. Such a simple and convenient approach could facilitate recognizing the entrapped isomers during separation; moreover, it can also contribute to real‐time monitoring separation processes. Furthermore, these materials could also be useful for pollutant removal and environmental monitoring.

Nonporous adaptive pillararene crystals are a new class of molecular‐level functional materials composed of a specific number of 1,4‐dialkoxy benzene units connected by methylene groups.^[^ ^14^ ^]^ Unlike traditional robust porous adsorbents, these pillararenes are nonporous in the initial state, but intrinsic porosity can be induced by certain vaporized species.^[^ ^15^ ^]^ These pillararenes exhibit excellent performances for the adsorption and separation of small organic isomers, including 1‐bromopropane/2‐bromopropane, 2‐chloropyridine/3‐chloropyridine, and p‐chlorotoluene/o‐chlorotoluene.^[^ ^3^, ^16^ ^]^ This behavior is mainly due to their adaptive structures, which facilitate the formation of strong pore‐inside intermolecular interactions, such as hydrogen bonding and *π*–*π* stacking interactions, with the entrapped isomers through guest‐induced structural transformations. As the resulting host–guest complexes have high thermodynamic stabilities, these pillararenes exhibit high selectivities for the target isomers.

Because of their electron‐rich characteristics, these pillararenes have also been used as donors with acceptors such as 1,2,4,5‐tetracyanobenzene and *N*,*N*′‐bis(n‐butyl)pyromellitic diimide to form exo‐wall cocrystals.^[^ ^17^ ^]^ In such cocrystals, the acceptor resides outside the pillararene and forms strong pore‐outside charge‐transfer (CT) interactions with adjacent pillararenes. Upon simple exposure to organic vapors, these cocrystals display vapochromic properties because the CT interactions can be easily altered by transformations of these adaptive structures triggered by guest vapors, thus leading to an obvious change in color. Furthermore, the vapochromic properties can be easily modulated by introducing different acceptors. In spite of these advantages, current pillararene‐based cocrystals exhibit attractive recognition properties mainly to single organic compound such as CH_2_Cl_2_, CHCl_3_, n‐propanal, and n‐butanal or organic mixtures with obvious structural differences.^[^ ^17^ ^]^ By contrast, for organic isomers, these cocrystals still suffer from poor vapochromic selectivity, as their CT interactions are not strong enough to distinguish minor structural differences between isomers. Moreover, most studies about vapochromic materials have focused on trial‐and‐error measurements demonstrating their vapochromic behaviors, with little development of an effective strategy for the design of suitable vapochromic materials according to target guests.

Here, we propose an effective strategy for the simultaneous recognition and separation of organic isomers using pillararene‐based CT cocrystals through the cooperative control of pore‐inside and pore‐outside intermolecular interactions. In this strategy (Figure 1b), we envisioned that it would be critical to design suitable pillararene‐based cocrystals based on the structures of the target isomers. The cocrystals should meet the following requirements: 1) The pillararene (donor) should have stronger pore‐inside interactions with the target isomer than with competing isomers when the isomers are loaded within the pores of the pillararene. 2) After cocrystal formation with the acceptor, the strong pore‐inside interactions should be maintained, resulting in high thermodynamic stability upon adsorption of the target isomer by the cocrystal and thus achieving selectivity for the target isomer. 3) When the competing isomers are loaded into the cocrystals, the pore‐outside CT interactions should be almost unchanged, thus maintaining the original color of the cocrystals. By contrast, when the target isomers are loaded, the CT interactions should be greatly weakened or strengthened, resulting in an obvious color change, thus achieving selective recognition of the target isomer. We anticipated that cooperative control of the pore‐inside and pore‐outside intermolecular interactions could endow the cocrystals with high selectivity for the simultaneous recognition and separation of target isomers. Consequently, the proposed strategy is simpler and more convenient than traditional two‐step strategies.

## 2 Results and Discussion

**Figure 2:**
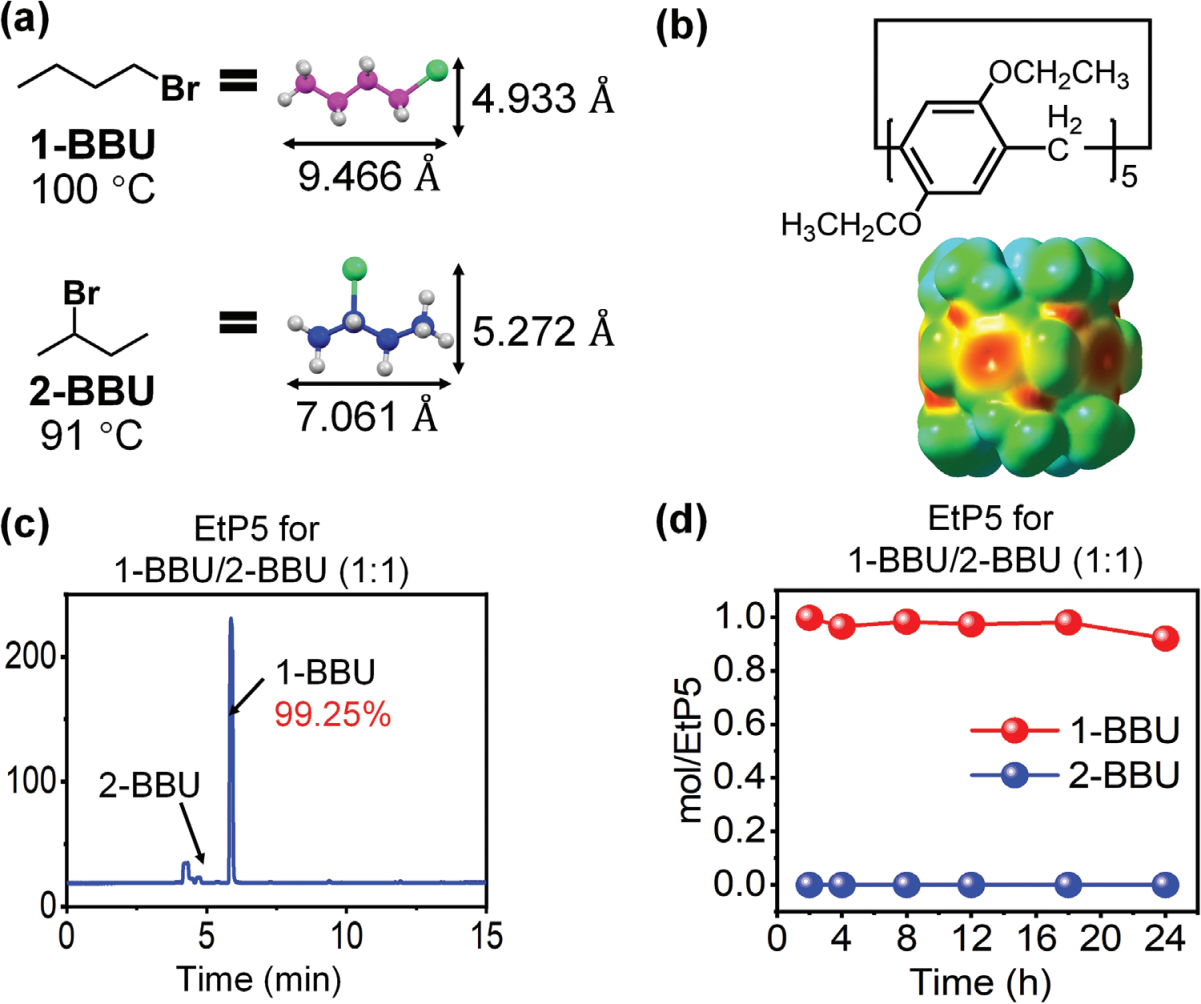
Properties of 1‐BBU/2‐BBU and EtP5. a) Chemical structures and boiling points of 1‐BBU and 2‐BBU. b) Chemical structure and electrostatic potential surface of EtP5. c) Sensitivity of EtP5 to 1‐BBU:2‐BBU = 1:1 v/v, as measured by gas chromatography. d) Adsorption curves of EtP5 with a mixed vapor of 1‐BBU:2‐BBU = 1:1 v/v, as measured by ^1^H NMR spectroscopy.

To confirm the viability of the proposed strategy, 1‐bromobutane (1‐BBU) was chosen as a typical example because it is an important hydrocarbon reagent and raw material in organic synthesis.^[^ ^18^ ^]^ However, as 1‐BBU is mainly produced by the bromination of butane or 1‐butene, it is often obtained as a mixture with its branched isomer (2‐bromobutane; 2‐BBU). These isomers have similar molecular sizes and properties (e.g., boiling point: 100 °C for 1‐BBU vs 91 °C for 2‐BBU; **Figure** **2**a; and Figure S1, Supporting Information). Moreover, they are highly volatile and toxic to humans.^[^ ^13^ ^]^ Therefore, it is necessary to recognize and separate 1‐BBU from its competing isomer. Although several pillararenes have exhibited excellent separation performance for haloalkane isomers, including bromoalkane isomers,^[^ ^16^, ^18^ ^]^ the simultaneous recognition and separation of these isomers have not been reported.

### 2.1 Pillararene Selection

To achieve simultaneous recognition and separation, a suitable pillararene and a matching acceptor should be chosen to construct a cocrystal that meets the above requirements. Based on the structure of 1‐BBU, a nonporous perethylated pillar[5]arene (denoted EtP5) was chosen because its *π*‐electron‐rich wall (Figure 2b) can facilitate electron donation.^[^ ^19^ ^]^ Moreover, EtP5 exhibits excellent performance for the adsorption and separation of 1‐chlorobutane (a 1‐BBU analog) from its competing isomer (2‐chlorobutane) because the pore‐inside intermolecular interactions with 1‐chlorobutane are stronger than those with its isomer.^[^ ^20^ ^]^ Thus, we anticipated that EtP5 would also form stronger pore‐inside intermolecular interactions with 1‐BBU than with 2‐BBU, leading to high selectivity for 1‐BBU.

**Figure 3:**
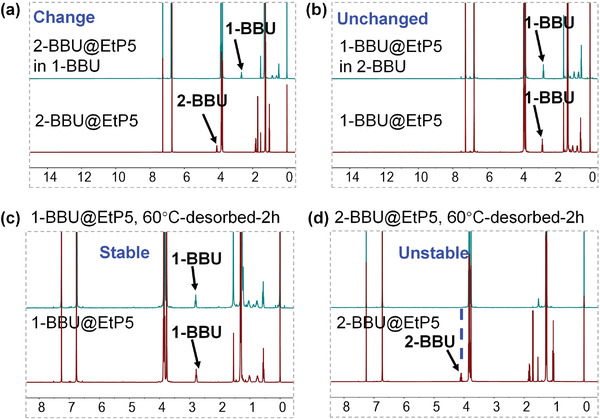
Guest exchange and desorption properties of 1‐BBU@EtP5 and 2‐BBU@EtP5. ^1^H NMR spectra of a) 2‐BBU@EtP5 and guest exchange with 1‐BBU after 8 h. b) 1‐BBU@EtP5 and guest exchange with 2‐BBU after 8 h. c) 1‐BBU@EtP5 and 1‐BBU@EtP5 after desorption for 2 h at 60 °C. d) 2‐BBU@EtP5 and 2‐BBU@EtP5 after desorption for 2 h at 60 °C.

**Figure 4:**
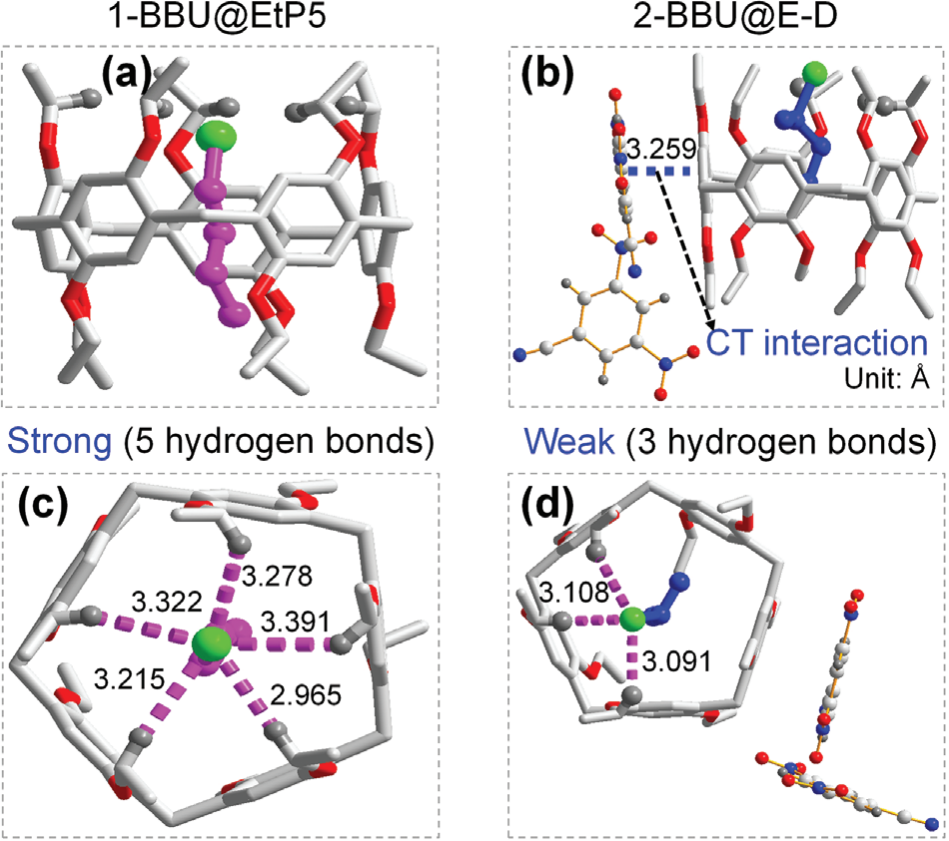
Crystal structures of cocrystal complexes. a,b) Crystal structures of 1‐BBU@EtP5 and 2‐BBU@E‐D. c,d) Pore‐inside hydrogen‐bonding interactions of 1‐BBU@EtP5 and 2‐BBU@E‐D. The purple dashed lines indicate the pore‐inside hydrogen bonds.

To confirm this assumption, the adsorption properties of EtP5 for 1‐BBU and 2‐BBU were investigated. Based on the ^1^H NMR spectra, the uptake amounts of 1‐BBU and 2‐BBU were calculated to be approximately one molecule per EtP5 (Figures S4 and S5 and Table S1, Supporting Information). The resulting powders (denoted 1‐BBU@EtP5 and 2‐BBU@EtP5) were the same color (white) as EtP5 itself (Figure S2, Supporting Information). Moreover, the powder X‐ray diffraction (PXRD) patterns were almost the same, indicating that these two powders had similar structures (Figure S6, Supporting Information). To explore the strength of the pore‐inside intermolecular interactions in 1‐BBU@EtP5 and 2‐BBU@EtP5, their thermodynamic stabilities were investigated through guest exchange and desorption. After exposing the 2‐BBU@EtP5 powder to 1‐BBU vapor at room temperature for 8 h, the resulting powder was characterized by ^1^H NMR spectroscopy. The ^1^H NMR spectrum showed the appearance of the characteristic signal of 1‐BBU and the disappearance of the 2‐BBU signal (**Figure** **3**a). The ^1^H NMR spectrum also revealed an ≈1:1 stoichiometry between 1‐BBU and EtP5. However, when the 1‐BBU@EtP5 powder was exposed to 2‐BBU vapor under the same conditions, the structure remained intact (Figure 3b). Even when the exchange time was extended to 72 h, the 1‐BBU@EtP5 complex was maintained (Figure S9, Supporting Information). Moreover, the differential scanning calorimetry (DSC)‐thermal gravimetry (TG) curves showed that the desorption temperature of 1‐BBU from EtP5 (140 °C) was remarkably higher than that of 2‐BBU (60 °C) (Figures S11 and S12, Supporting Information), although free 1‐BBU and 2‐BBU had similar boiling points. When 1‐BBU@EtP5 and 2‐BBU@EtP5 were separately heated at 60 °C for 2 h in an evacuated oven, the amount of 1‐BBU in 1‐BBU@EtP5 remained almost unchanged (Figure 3c), whereas the amount of 2‐BBU in 2‐BBU@EtP5 greatly decreased, reaching nearly zero (Figure 3d). Furthermore, single‐crystal X‐ray analysis revealed that 1‐BBU@EtP5 has a molecular composition of 1:1 EtP5:1‐BBU, with one 1‐BBU molecule located in the pore of an EtP5 molecule (**Figure** **4**a; and Figure S66, Supporting Information). In the pore, strong intermolecular hydrogen‐bonding interactions form between the Br atom of 1‐BBU and the C—H groups of EtP5. Each 1‐BBU molecule participates in five hydrogen bonds (C—H···Br) (Figure 4c), similar to those observed upon the adsorption of 1‐chlorobutane by EtP5.^[^ ^20^ ^]^ Thus, these results demonstrate that EtP5 has stronger pore‐inside intermolecular interactions with 1‐BBU than with 2‐BBU, resulting in a host–guest complex with higher thermodynamic stability.

To confirm the stronger pore‐inside intermolecular interactions between EtP5 and 1‐BBU, the pore‐inside binding energies of EtP5 and 1‐BBU or 2‐BBU were calculated by first‐principle density functional model. As shown in **Table** **1**, the calculated binding energy of EtP5 and 1‐BBU was −80.26 kJ mol^−1^, which is much higher than that for 2‐BBU, confirmed that EtP5 has stronger pore‐inside intermolecular interactions with 1‐BBU.

**Table 1:** DFT calculated data of 1‐BBU@EtP5 and 2‐BBU@EtP5

Entry	*E*_EtP5_ (hartree)	*E*_BBU_ (hartree)	*E*_complex_(hartree)	*E*_binding energy_ [hartree]^a)^	*E*_binding energy_ [kJ mol^−1^]^a)^
1‐BBU@EtP5	−2888.812	−2730.021	−5618.863 624	−0.030 623 536	−80.26
2‐BBU@EtP5	−2888.817	−2730.078	−5618.919 736	−0.024 736 155	−64.83

^a)^ The binding energy of EtP5 and BBU calculated by *E*_binding energy_ = *E*_complex_‐*E*_EtP5_‐*E*_BBU_.

In addition, the selective separation of 1‐BBU and 2‐BBU was also investigated (Figures S2 and S5, Supporting Information). Upon exposure to an equal‐volume two‐component 1‐BBU/2‐BBU mixture, EtP5 exhibited a high selectivity of 99.25% for 1‐BBU, as determined by gas chromatography (Figure 2c), further confirming the stronger pore‐inside intermolecular interactions between EtP5 and 1‐BBU. The uptake of 1‐BBU was similar to that for single‐component adsorption (Figure 2d). Because EtP5 had stronger pore‐inside intermolecular interactions with 1‐BBU and exhibited excellent separation performance, it was chosen as a donor for the construction of a suitable cocrystal.

### 2.2 Matching Acceptor Selection

**Figure 5:**
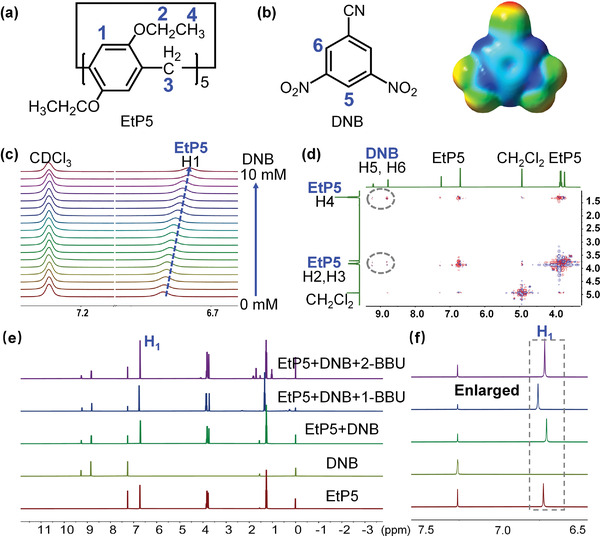
Donor–acceptor interactions. a) Chemical structure of EtP5. b) Chemical structure and electrostatic potential surface of DNB. c) Partial ^1^H NMR spectra of EtP5 (1.00 mm, 400 MHz) in the presence of various molar equivalents of DNB in CDCl_3_ at 293 K. d) 2D NOESY NMR spectrum (400 MHz, 298 K, CDCl_3_) of a solution of EtP5 (60 mm) and DNB (120 mm). e) ^1^H NMR spectra and f) enlarged spectra of EtP5, DNB, EtP5+DNB, EtP5+DNB+1‐BBU, and EtP5+DNB+2‐BBU.

Based on the chosen donor, 3,5‐dinitrobenzonitrile (DNB) was strategically chosen as a matching acceptor because it contains three electron‐withdrawing groups and is a common electron‐deficient system (**Figure** **5**b; and Figure S14, Supporting Information).^[^ ^19b^ ^]^ Upon the gradual addition of DNB to a CDCl_3_ solution of EtP5, the H‐proton on the benzene ring of EtP5 (H1 of EtP5) gradually moved upfield in the ^1^H NMR spectrum (Figure 5c).^[^ ^17^, ^21^ ^]^ In addition, correlation signals were observed between the H protons on the EtP5 benzene ring (H2, H3, and H4 of EtP5) and the H protons on the DNB benzene ring (H5 and H6 of DNB) in their 2D NOESY spectrum (Figure 5d). These observations indicate the presence of strong interactions between EtP5 and DNB in solution, which are expected to facilitate cocrystal formation.^[^ ^19a^ ^]^

More importantly, when 2‐BBU was added to a CDCl_3_ solution of EtP5 and DNB (denoted EtP5+DNB+2‐BBU), the H1 signal of EtP5 (≈6.70 ppm, Figure 5f) was consistent with that observed for the original solution (EtP5+DNB). However, when the same amount of 1‐BBU was added to a CDCl_3_ solution of EtP5 and DNB (denoted EtP5+DNB+1‐BBU), the H1 signal of EtP5 shifted downfield, similar to that observed for free EtP5. These results indicate that the interactions between EtP5 and DNB remained intact upon 2‐BBU addition, but these interactions were almost completely destroyed upon 1‐BBU addition. Thus, the combination of EtP5 and DNB can distinguish minor differences between 1‐BBU and 2‐BBU.

Further, to predict pore‐outside intermolecular interactions, we calculated the intermolecular pore‐outside binding energies of DNB and EtP5 (DNB/EtP5), DNB and 2‐BBU@EtP5 (DNB/2‐BBU@EtP5), and DNB and 1‐BBU@EtP5 (DNB/1‐BBU@EtP5) using the DFT‐D3 method (density functional theory incorporating the D3 version of Grimme's dispersion) at the B3LYP/6‐31G level (Figures S14 and S15, Supporting Information).^[^ ^22^ ^]^ The pore‐outside binding energy for DNB/EtP5 (−107.05 kJ mol^−1^) was similar to that for DNB/2‐BBU@EtP5 (−109.00 kJ mol^−1^). However, the binding energy for DNB/1‐BBU@EtP5 was considerably higher (−95.67 kJ mol^−1^), demonstrating that both DNB/EtP5 and DNB/2‐BBU@EtP5 have stronger pore‐outside intermolecular interactions than DNB/1‐BBU@EtP5. Thus, the introduction of 1‐BBU or 2‐BBU into a mixture of EtP5 and DNB could result in different strengths of pore‐outside intermolecular interactions between EtP5 and DNB, which could facilitate distinguishing between 1‐BBU and 2‐BBU. Based on these experimental and calculation results, DNB was selected as the matching acceptor.

### 2.3 Synthesis and Characterization of Cocrystals

**Figure 6:**
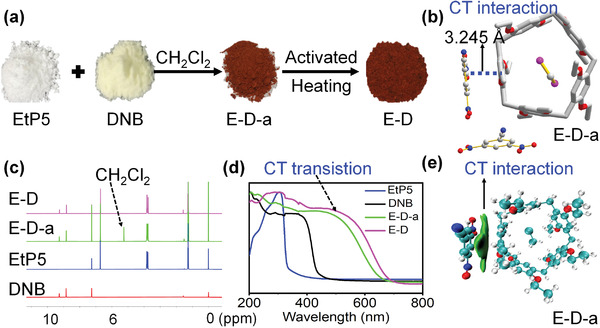
Characteristics of E‐D cocrystal. a) Preparation of E‐D cocrystal and color changes. b) Crystal structure of E‐D‐a. c) ^1^H NMR spectra of DNB, EtP5, E‐D‐a, and E‐D. d) Normalized solid‐state UV–vis spectra of DNB, EtP5, E‐D‐a, and E‐D. e) Host–guest interactions of E‐D‐a.

Using the chosen donor and matching acceptor, the preparation of cocrystals was explored. DNB was added to a CH_2_Cl_2_ solution of EtP5 at room temperature. After 3 days, several red crystals (denoted E‐D‐a) were obtained, which had a completely different color compared to the precursors (**Figure** **6**a). The infrared spectrum of E‐D‐a showed typical absorption peaks at 2200 and 2900 cm^−1^ associated with the CN group of DNB and CH_2_ groups of EtP5 (Figure S16, Supporting Information). The ^1^H NMR spectrum also indicated that E‐D‐a contained EtP5 and DNB, with a stoichiometry of 1:2 (Figure 6c; and Figure S17, Supporting Information). Moreover, the solid‐state UV–vis spectrum exhibited an obvious broad band at ≈500 nm (Figure 6d), which was not observed in the spectrum of either EtP5 or DNB. This band was assigned as a characteristic absorption of the pore‐outside CT complexation between EtP5 and DNB in E‐D‐a.^[^ ^23^ ^]^ Single‐crystal X‐ray diffraction measurements were preformed to further confirm the structure of E‐D‐a and its pore‐outside CT intermolecular interactions. The E‐D‐a structure was revealed to have a molecular composition of 1:2:2 EtP5:DNB:CH_2_Cl_2_. A CH_2_Cl_2_ molecule was located in the pore of the EtP5 molecule. Each DNB molecule was located outside the EtP5 cavity, arranged parallel to a benzene ring of EtP5. A strong *π*–*π* interaction with an interplanar distance of only 3.245 Å was formed, further demonstrating that the strong pore‐outside CT interaction between EtP5 and DNB in E‐D‐a (Figure 6b; and Figure S60, Supporting Information). These CT interactions were also confirmed by noncovalent interaction analysis using the Gaussian 09 and Multiwfn programs (Figure 6e; and Figure S61, Supporting Information).^[^ ^24^ ^]^

The E‐D‐a crystals were activated by heating at 130 °C for 30 min to remove CH_2_Cl_2_ (denoted E‐D). ^1^H NMR spectroscopy and thermogravimetric analysis (TGA) confirmed the complete removal of CH_2_Cl_2_, resulting in an EtP5:DNB ratio of 1:2 (Figure 6c; and Figures S19 and S20, Supporting Information). E‐D maintained the original red color. In addition, the solid‐state UV–vis spectrum exhibited an obvious broad band at ≈500 nm, similar to that of E‐D‐a, indicating that the strong pore‐outside CT interaction between EtP5 and DNB remained unchanged. The PXRD pattern of E‐D confirmed its crystalline nature and was similar to that of E‐D‐a, indicating that the cocrystal structure was maintained (Figure S21, Supporting Information).

### 2.4 Adsorption of Single‐Component Bromoalkane Isomers

**Figure 7:**
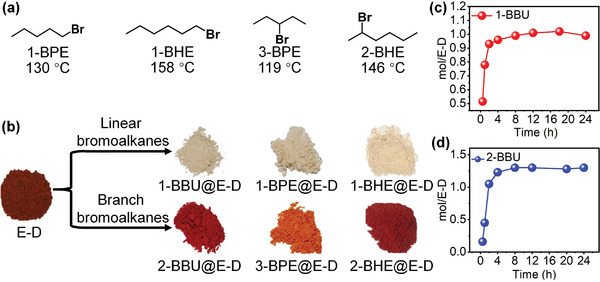
Adsorption of various bromoalkene isomers. a) Other bromoalkanes and their boiling points. b) Color changes upon exposure of the E‐D cocrystal powder to linear and branched bromoalkanes. c,d) Adsorption curves of E‐D cocrystal with individual isomers (1‐BBU or 2‐BBU), as measured by ^1^H NMR spectroscopy.

The adsorption behavior of E‐D for 1‐BBU and 2‐BBU was investigated. Surprisingly, the exposure of E‐D to 1‐BBU vapor for 50 min resulted in a distinct color change from red to white (**Figure** **7**b; and Figure S25, Supporting Information), which was easily observed with the naked eye. After 4 h, the uptake amount of 1‐BBU reached saturation at nearly one molecule per E‐D molecule (Figure 7c), which is consistent with that observed for EtP5. By contrast, when E‐D was exposed to 2‐BBU vapor, no change in color alternation occurred (Figure 7b; and Figure S28, Supporting Information). However, the uptake amount of 2‐BBU was nearly one molecule per E‐D molecule (Figure 7d). The same color change was observed upon exposure of E‐D to the vapor of other linear bromoalkanes, such as 1‐bromopentane (1‐BPE, Figure 7a) and 1‐bromohexane (1‐BHE), whereas the red color was retained when E‐D was exposed to their branched isomers, such as 3‐bromopentane (3‐BPE) and 2‐bromohexane (2‐BHE). The uptake amounts were also nearly one molecule per E‐D molecule, except for 2‐BHE (≈0.696 mol 2‐BHE per mol E‐D, Figure S34, Supporting Information). The resultant materials are denoted 1‐BBU@E‐D, 1‐BPE@E‐D, 1‐BHE@E‐D, 2‐BBU@E‐D, 3‐BPE@E‐D, and 2‐BHE@E‐D. By contrast, when free EtP5 adsorbed these linear or branched bromoalkanes, all the products maintained the original white color (Figure S2, Supporting Information).

### 2.5 Intermolecular Interactions

The structures of 1‐BBU@E‐D and 2‐BBU@E‐D as well as their pore‐outside and pore‐inside intermolecular interactions were investigated using various methods.

#### 2.5.1 Pore‐Outside CT Interactions

**Figure 8:**
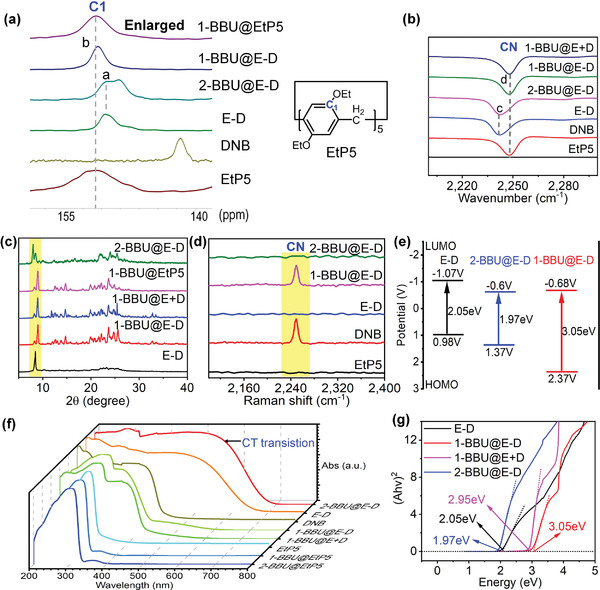
Characterization of pore‐outside CT interactions. a) Enlarged solid‐state ^13^C NMR spectra of EtP5, DNB, E‐D, 2‐BBU@E‐D, 1‐BBU@E‐D, and 1‐BBU@EtP5. b) Partial FT‐IR spectra of EtP5, DNB, E‐D, 2‐BBU@E‐D, 1‐BBU@E‐D, and 1‐BBU@E+D. c) Partial PXRD patterns of E‐D, 1‐BBU@E‐D, 1‐BBU@E+D, 1‐BBU@EtP5, and 2‐BBU@E‐D. d) Enlarged Raman spectra of EtP5, DNB, E‐D, 1‐BBU@E‐D, and 2‐BBU@E‐D. e) Calculated bandgaps of E‐D, 1‐BBU@E‐D, and 2‐BBU@E‐D. f) Solid‐state UV–vis spectra of 2‐BBU@EtP5, 1‐BBU@EtP5, EtP5, 1‐BBU@E+D, 1‐BBU@E‐D, DNB, E‐D, and 2‐BBU@E‐D. g) Experimental bandgaps of E‐D, 1‐BBU@E‐D, 1‐BBU@E+D, and 2‐BBU@E‐D.

In the solid‐state ^13^C NMR spectra, the C1 signal of EtP5 (a peak, ≈150 ppm) in 2‐BBU@E‐D was the same as that observed for E‐D (**Figure** **8**a; and Figure S38, Supporting Information). By contrast, the C1 signal (b peak) in 1‐BBU@E‐D shifted downfield, similar to those of free EtP5 and 1‐BBU@EtP5.^[^ ^25^ ^]^ Further, in the IR spectra, a typical absorption peak associated with the CN group of DNB (c peak, ≈2250 cm^−1^) was observed for both 2‐BBU@E‐D and E‐D (Figure 8b; and Figure S39, Supporting Information).^[^ ^26^ ^]^ However, the corresponding absorption peak (d peak) in 1‐BBU@E‐D was redshifted, similar to those of free DNB and the physical mixture of 1‐BBU@EtP5 and DNB (denoted 1‐BBU@E+D). Moreover, the PXRD pattern of 2‐BBU@E‐D was similar to that of E‐D (Figure 8c). However, the PXRD pattern of 1‐BBU@E‐D differed from that of E‐D but was identical to those of 1‐BBU@EtP5 and 1‐BBU@E+D (Figure S40, Supporting Information). These results demonstrate that after 2‐BBU adsorption, the structure of E‐D did not change significantly. However, upon the adsorption of 1‐BBU, the E‐D structure underwent obvious changes, becoming similar to those of 1‐BBU@EtP5 and 1‐BBU@E+D.

Raman spectroscopy was used to further demonstrate the differences between the structures of 1‐BBU@E‐D and 2‐BBU@E‐D.^[^ ^25^, ^27^ ^]^ In the Raman spectrum of E‐D, the typical absorption peak (around 2240 cm^−1^) associated with the CN group of DNB completely disappeared (Figure 8d; and Figure S41, Supporting Information), possibly because the strong pore‐outside CT interactions between EtP5 and DNB in E‐D resulted in a shielding effect. Similarly, the typical absorption peak associated with the CN group was absent from the Raman spectrum of 2‐BBU@E‐D, indicating that the strong pore‐outside CT interactions between EtP5 and DNB in E‐D were maintained upon 2‐BBU adsorption. By contrast, the Raman spectrum of 1‐BBU@E‐D showed a strong absorption peak associated with the CN group, consistent with that of free DNB, demonstrating that 1‐BBU adsorption by E‐D greatly weakened the CT interaction, resulting in the disappearance of the shielding effect.

The pore‐outside CT interactions in 1‐BBU@E‐D and 2‐BBU@E‐D were also investigated using solid‐state UV–vis spectroscopy. The solid‐state UV–vis spectrum of 2‐BBU@E‐D (Figure 8f) exhibited an obvious broad adsorption band at ≈500 nm, similar to that observed for E‐D. However, this adsorption band disappeared completely in the spectrum of 1‐BBU@E‐D. The adsorption bands of 1‐BBU@E‐D can be regarded as the sum of those of pure EtP5 and DNB, as also observed for 1‐BBU@E+D. These results indicate that the pore‐outside CT interactions of E‐D remain unchanged after 2‐BBU adsorption.^[^ ^23^ ^]^ However, after 1‐BBU adsorption by E‐D, the CT interactions were destroyed.

**Figure 9:**
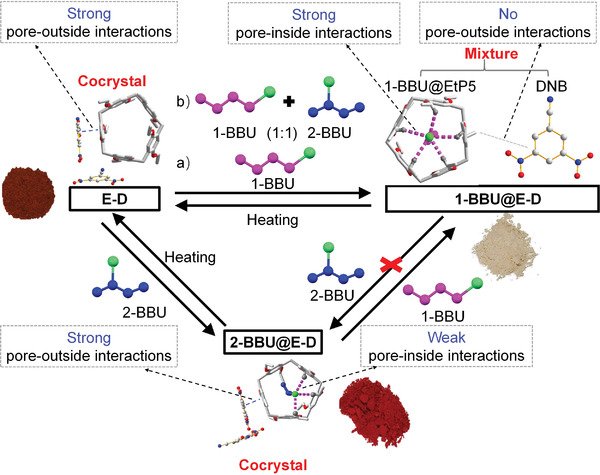
Color exchange mechanism between E‐D, 1‐BBU@E‐D, and 2‐BBU@E‐D.

Narrower bandgaps (*E*_g_) are often associated with stronger CT interactions.^[^ ^28^ ^]^ Therefore, the *E*_g_ values of 2‐BBU@E‐D and 1‐BBU@E‐D were investigated to clarify the strengths of the pore‐outside CT interactions. As a reference, the *E*_g_ value of E‐D was also explored. First, the positions of the highest occupied molecular orbital (HOMO) was determined using ultraviolet photoelectron spectroscopy (UPS).^[^ ^28^ ^]^ By subtracting the width of the UPS spectrum from the excitation energy (eV), the positions of the HOMO for E‐D, 2‐BBU@E‐D, and 1‐BBU@E‐D were calculated as 0.98, 1.37, and 2.37 V (vs NHE, pH 7, Figure 8e; and Figures S42–S48, Supporting Information), respectively. The semiconductor band structures were determined using Mott–Schottky tests at frequencies of 500, 1000, and 1500 Hz. The obtained C^−2^ curves intersected the *x*‐axis at −1.27 (E‐D), −0.8 (2‐BBU@E‐D), −0.88 (1‐BBU@E‐D) V versus Ag/AgCl, respectively.^[^ ^28^, ^29^ ^]^ The lowest unoccupied molecular orbital (LUMO) positions were calculated as −1.07 (E‐D), −0.6 (2‐BBU@E‐D), −0.68 (1‐BBU@E‐D) V (vs NHE, pH 7). Based on the positions of the HOMO and LUMO, the *E*_g_ value of 2‐BBU@E‐D was calculated as 1.97 eV, which is very close to that of E‐D (2.05 eV) but much smaller than that of 1‐BBU@E‐D (3.05 eV). The *E*_g_ value of 1‐BBU@E‐D was similar that of the physical mixture of 1‐BBU@EtP5 and DNB (1‐BBU@E+D, 2.95 eV). All the *E*_g_ values were also verified based on UV–vis data using the Kubelka–Munk formula (Figure 8g; and Figure S49, Supporting Information), which is a common method for evaluating the bandgap of a material.^[^ ^30^ ^]^ Therefore, upon adsorption of 2‐BBU, the structure of E‐D remained almost unchanged and the resultant material (2‐BBU@E‐D) maintained a narrow bandgap. Consequently, charge transfer was facilitated, endowing 2‐BBU@E‐D with strong pore‐outside CT interactions between EtP5 and DNB. Thus, 2‐BBU@E‐D maintained the original red color. However, 1‐BBU adsorption by E‐D resulted in considerably widening of the bandgap, thus hindering charge transfer. As a result, the pore‐outside CT interactions between EtP5 and DNB disappeared. Owing to the absence of CT interactions, EtP5 was completely separated from DNB in 1‐BBU@E‐D, forming a physical mixture of 1‐BBU@E‐D and DNB (**Figure** **9**). Thus, the color of the mixture changed from red to white. These results clearly show that 2‐BBU@E‐D and E‐D had strong pore‐outside CT interactions, whereas 1‐BBU@E‐D did not (Figure 9).

#### 2.5.2 Pore‐Inside Intermolecular Interaction

**Figure 10:**
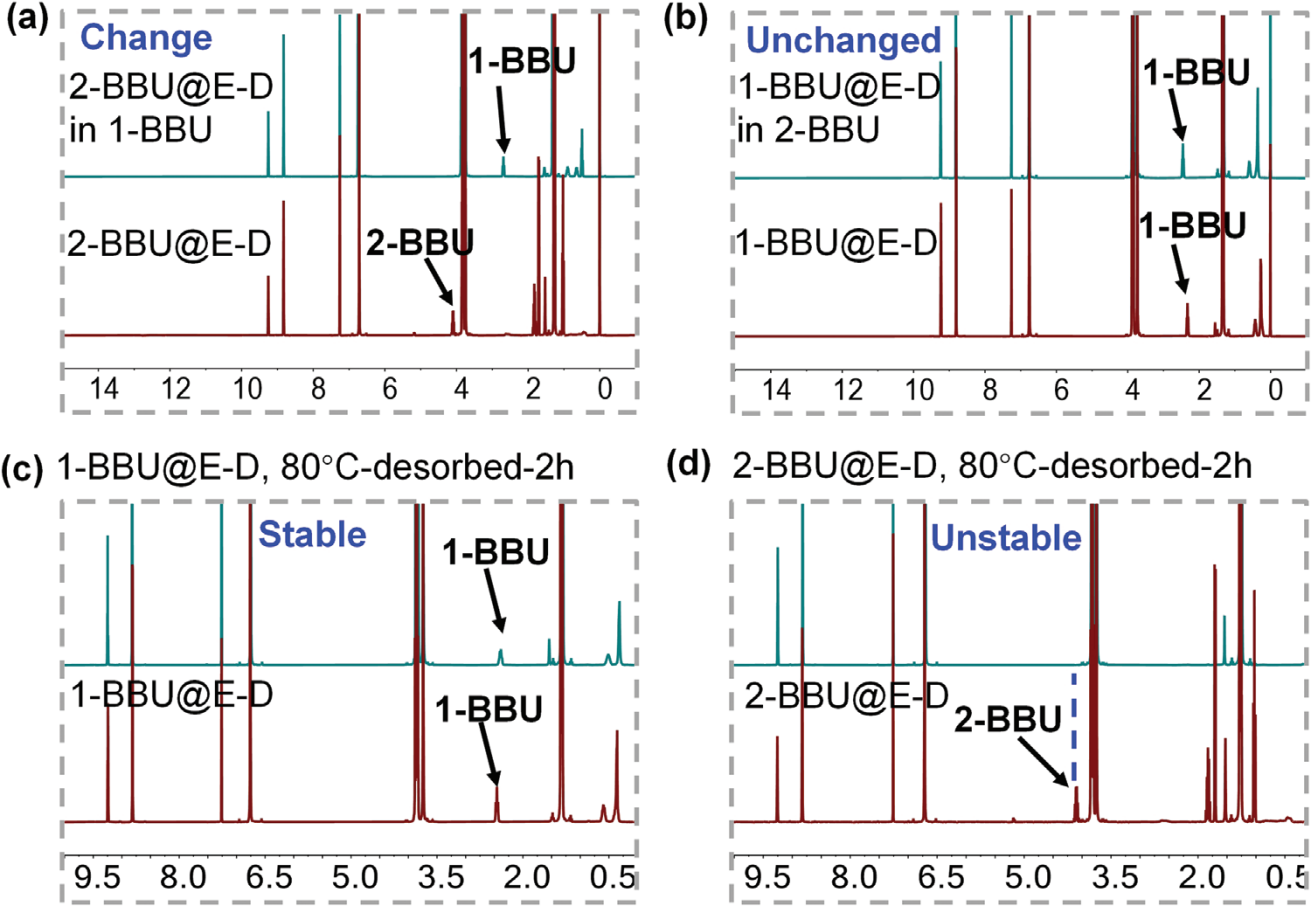
Guest exchange and desorption by 1‐BBU@E‐D and 2‐BBU@E‐D. ^1^H NMR spectra of a) 2‐BBU@E‐D and guest exchange with 1‐BBU after 8 h. b) 1‐BBU@E‐D and guest exchange with 2‐BBU after 8 h. c) 1‐BBU@E‐D and 1‐BBU@E‐D after desorption for 2 h at 80 °C. d) 2‐BBU@E‐D and 2‐BBU@E‐D after desorption for 2 h at 80 °C.

To explore the pore‐inside intermolecular interactions in 2‐BBU@E‐D and 1‐BBU@E‐D, the thermodynamic stabilities were investigated through guest exchange and desorption. The operation processes were very similar to those for 2‐BBU@EtP5 and 1‐BBU@EtP5. After exposure of the 2‐BBU@E‐D powder to 1‐BBU vapor at room temperature for 8 h, the color of the powder changed from red to white (Figure 9; and Figure S51, Supporting Information). ^1^H NMR and PXRD results showed that the resultant material was different from the precursor (2‐BBU@E‐D) but identical to 1‐BBU@E‐D (**Figure** **10**a; and Figure S52, Supporting Information). However, when the 1‐BBU@E‐D powders was exposed to 2‐BBU vapor under the same conditions, the white color did not change (Figure 9) and the structure remained intact, as revealed by ^1^H NMR spectroscopy and PXRD (Figure 10b; and Figure S52, Supporting Information). Moreover, the DSC‐TG curves showed that the desorption temperature of 1‐BBU from E‐D (130 °C) was considerable higher than that of 2‐BBU (80 °C) (Figures S54 and S55, Supporting Information). When 1‐BBU@E‐D and 2‐BBU@E‐D were separately heated in an evacuated oven at 80 °C for 2 h, ^1^H NMR spectroscopy revealed that the amount of 1‐BBU in 1‐BBU@E‐D remained almost unchanged, whereas 2‐BBU completely disappeared from 2‐BBU@E‐D (Figure 10c,d). For comparison, the thermodynamic stability of 1‐BBU@E+D was also explored because the adsorption of 1‐BBU by E‐D could form a mixture of 1‐BBU@EtP5 and DNB. When 1‐BBU@E+D was separately exposed to 2‐BBU vapor at room temperature, the structure remained intact (Figures S57 and S58, Supporting Information). Moreover, the desorption temperature of 1‐BBU@E+D reached 140 °C (Figure S59, Supporting Information). These results demonstrate that E‐D had stronger pore‐inside interactions with 1‐BBU than with 2‐BBU, resulting in 1‐BBU@E‐D with a higher thermodynamic stability.

To further demonstrate the strengths of pore‐outside and pore‐inside intermolecular interactions in 1‐BBU@E‐D and 2‐BBU@E‐D, we attempted to obtain both single crystals of 1‐BBU@E‐D and 2‐BBU@E‐D and used X‐ray crystallography to directly observe their molecular interactions. Under the conditions for E‐D‐a crystal formation, 2‐BBU was added to a mixture of EtP5 and DNB in CH_2_Cl_2_. After 5 days, red crystals were obtained, which were confirmed as 2‐BBU@E‐D using ^1^H NMR spectroscopy and PXRD (Figures S64 and S65, Supporting Information). However, under the same conditions, 1‐BBU@E‐D crystals were not produced, although a few white 1‐BBU@EtP5 crystals were formed. Despite numerous attempts, 1‐BBU@E‐D crystals could not be obtained. It is possible that the CT interactions between EtP5 and DNB were destroyed after 1‐BBU was added to the solution, as indicated by ^1^H NMR spectroscopy (Figure 5f). The resulting large separation between EtP5 and DNB likely hindered the formation of 1‐BBU@E‐D crystals.

Single‐crystal X‐ray analysis revealed that 2‐BBU@E‐D contained one EtP5 molecule and two DNB molecules per unit cell. A 2‐BBU molecule was located in the pore of the EtP5 molecule (Figure 4b; and Figure S62, Supporting Information). Every DNB molecule was arranged outside the EtP5 cavity and arranged parallel to a benzene ring of EtP5. A strong *π*–*π* interaction with an interplanar distance of 3.259 Å was formed, which very similar to that observed for E‐D‐a (3.245 Å), further confirming that E‐D maintained strong pore‐outside CT interactions between EtP5 and DNB after 2‐BBU adsorption (Figure S63, Supporting Information).

The most significant difference between 2‐BBU@E‐D and 1‐BBU@EtP5 was the number of hydrogen bonds (C—H···Br) between the Br atoms of bromobutanes (2‐BBU and 1‐BBU) and the C—H group of EtP5 in the pores of EtP5 (Figure 4c,d). In 2‐BBU@E‐D, each 2‐BBU molecule participated in only three hydrogen bonds (C—H···Br). By contrast, each 1‐BBU molecule participated in more than five hydrogen bonds (C—H···Br). Thus, the pore‐inside hydrogen‐bonding interactions in 1‐BBU@EtP5 were stronger. The PXRD pattern of E‐D after the adsorption of 2‐BBU matched well with that of the simulated guest‐loaded single‐crystal structure (Figure S64, Supporting Information). Moreover, the PXRD pattern of E‐D after the adsorption of 1‐BBU was also consistent with the simulated single‐crystal data of 1‐BBU@EtP5 (Figure S68, Supporting Information), further indicating that E‐D could form a mixture of 1‐BBU@EtP5 and DNB upon 1‐BBU adsorption. Therefore, similar to free EtP5, EtP5 in the E‐D cocrystal still maintained stronger pore‐inside interactions with 1‐BBU than with 2‐BBU.

### 2.6 Recognition and Separation of E‐D for Bromoalkane Isomers in Two‐Component Mixtures

**Figure 11:**
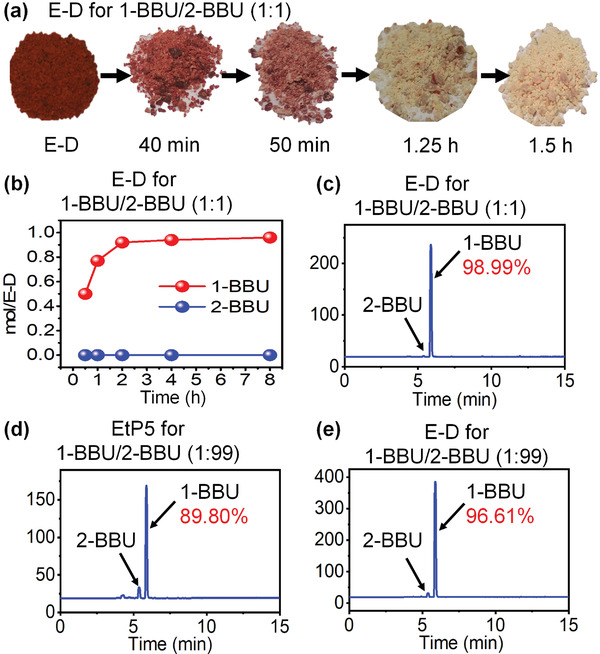
Separation of bromoalkane isomers. a) Color changes of E‐D upon exposure to 1‐BBU:2‐BBU = 1:1 v/v. b) Adsorption curves of E‐D with a mixed vapor of 1‐BBU:2‐BBU = 1:1 v/v, as measured by ^1^H NMR spectroscopy. c) Selectivity of E‐D for 1‐BBU:2‐BBU = 1:1 v/v, as measured by gas chromatography. d,e) Selectivity of EtP5 and E‐D for 1‐BBU:2‐BBU = 1:99 v/v, as measured by gas chromatography.

The stronger pore‐inside interactions and the absence of pore‐outside CT interactions in 1‐BBU@E‐D motivated us to examine the competitive recognition and separation of 1‐BBU and 2‐BBU using E‐D. Upon exposure to an equal‐volume two‐component mixture of 1‐BBU and 2‐BBU, the color of E‐D changed from red to white after 1.5 h (**Figure** **11**a), and the resultant solid was identified as 1‐BBU@E‐D by PXRD (Figure S70, Supporting Information). Further, ^1^H NMR and gas chromatography analyses indicated that the selectivity for 1‐BBU reached 98.99% (Figure 11c). In addition, the uptake amount was similar to that observed during single‐component sorption (Figure S73 and Table S3, Supporting Information). Surprisingly, even when the two‐component mixture only contained trace 1‐BBU (1:99 1‐BBU:2‐BBU), the color of E‐D changed from red to white after 6 h. In this case, the selectivity of E‐D for 1‐BBU was 96.61% (Figure 11e), which is remarkably higher than that of free EtP5 (89.80%, Figure 11d). Moreover, the recognition and separation of E‐D in the equal‐volume two‐component mixture were monitored over time by PXRD (Figure S70, Supporting Information) and ^1^H NMR (Figure 11b). Throughout the entire adsorption process, 1‐BBU@E‐D was the only product formed, further confirming the high selectivity for 1‐BBU. As expected, the stronger pore‐inside interactions and the absence of pore‐outside CT interactions in 1‐BBU@E‐D resulted in extremely high selectivity for the recognition and separation of 1‐BBU. These results confirm the feasibility of the proposed strategy.

To demonstrate the generality of this strategy, the recognition and separation of two additional equal‐volume two‐component mixtures of other bromoalkane isomers (1‐BPE/3‐BPE and 1‐BHE/2‐BHE) were investigated. When E‐D was exposed to either of these mixtures, the linear bromoalkane (1‐BPE or 1‐BHE) was adsorbed preferentially and the color changed from red to white (Figures S75 and S78, Supporting Information), as observed with 1‐BBU. Moreover, following absorption, these complexes exhibited excellent weathering resistance with no obvious loss of the encapsulated linear bromoalkanes. The color change was maintained, even after weathering over 1 month at room temperature, indicating the highly stable storage of linear bromoalkanes (Figures S81–S83, Supporting Information). Therefore, the proposed cooperative control strategy has considerable potential for the simultaneous recognition and separation of organic isomers.

### 2.7 Reversible and Cyclic

**Figure 12:**
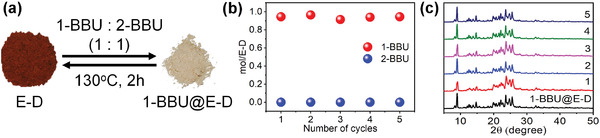
Reversibility and recycling performance. a) Reversible color change between E‐D and 1‐BBU@E‐D. b) Selectivity of E‐D over five cycles. c) PXRD patterns of 1‐BBU@E‐D over five cycles.

In practical production processes, reversibility and recycling performance are vital parameters. The removal of 1‐BBU absorbed in E‐D was achieved by heating at 130 °C, and the obtained solid product was identified as E‐D by PXRD, ^1^H NMR, and IR analyses (Figures S84–S87, Supporting Information). The recovered E‐D regained its original red color (**Figure** **12**a). Upon subsequent exposure of this red solid to 1‐BBU, a white solid was formed. The reversible color change could be repeated at least five times without discernible changes in the behavior. Furthermore, the recovered E‐D maintained its high selectivity for 1‐BBU in isomeric mixtures without degradation for five cycles (Figure 12b,c).

## 3 Conclusion

A simple and convenient strategy for the simultaneous recognition and separation of organic isomers was developed based on the cooperative control of pore‐outside and pore‐inside interactions. EtP5 and DNB were chosen as suitable donors and acceptors for distinguishing the minor difference between 1‐BBU and its isomer (2‐BBU). The corresponding cocrystal exhibited stronger pore‐inside interactions and much weaker pore‐outside CT interactions with 1‐BBU than with 2‐BBU. Consequently, the cocrystal exhibited a selectivity of almost 100% for 1‐BBU in equal‐volume two‐component mixtures. Even in two‐component mixtures containing trace 1‐BBU, the 1‐BBU selectivity of the cocrystal (96.61%) was nearly 10% higher than that of free EtP5. The preference for linear bromoalkanes was retained with two other two‐component mixtures (1‐BPE/3‐BPE and 1‐BHE/2‐BHE), thereby demonstrating the generality of this strategy. The selective adsorption of the linear bromoalkanes resulted in a color change from red to white, which was easily recognized by the naked eye. Moreover, the recognition and adsorption process was reversible, allowing the cocrystal to be recycled. This study provides new insights into the recognition and separation of organic isomers. We anticipate that the proposed strategy will be useful for the simultaneous recognition and separation of challenging isomers, such as xylenes, which are difficult or unfeasible through traditional methods.

## Conflict of Interest

The authors declare no conflict of interest.

## Supporting information

Supporting Information

## Data Availability

The data that support the findings of this study are available in the supplementary material of this article.
