# Supplementary material for: Simultaneous Recognition and Separation of Organic Isomers Via Cooperative Control of Pore‐Inside and Pore‐Outside Interactions
Source: Adv Sci (Weinh). 2022 Oct 28;9(36):2204963. doi: 10.1002/advs.202204963 (PMC9798982; doi:10.1002/advs.202204963)
Supplement: Supplementary file 1 — Supporting Information [file ADVS-9-2204963-s001.pdf]

ADVANCED SCIENCE
Open Access

Supporting Information

for *Adv. Sci.*, DOI 10.1002/advs.202204963

Simultaneous Recognition and Separation of Organic Isomers Via Cooperative Control of Pore-Inside and Pore-Outside Interactions

Shaomin Xue, Yujia Rong, Ning Ding, Chaofeng Zhao, Qi Sun, Shenghua Li and Siping Pang**

Supplementary Materials for

Simultaneous recognition and separation of organic isomers via cooperative control of pore-inside and pore-outside interactions

Shaomin Xue, Yujia Rong, Ning Ding, Chaofeng Zhao, Qi Sun, Shenghua Li and Siping Pang**

Dr. S. Xue, Dr. Y. Rong, Dr. N. Ding, Dr. C. Zhao, Dr. Q. Sun, Prof. S. Li, Prof. S. Pang
School of Materials Science & Engineering, Beijing Institute of Technology, 100081, P. R. China
E-mail: lishenghua@bit.edu.cn; pangsp@bit.edu.cn

Prof. S. Li
Yangtze Delta Region Academy, Beijing Institute of Technology, Jiaxing 314019, P. R. China

Contents

1. Materials
2. Methods
3. Choosing a suitable pillararene
4. Choosing a matching acceptor
5. Synthesis and characterization of cocrystal
6. E-D cocrystal adsorption single component bromoalkane
7. Investigation of intermolecular interactions
8. Crystal data and structures
9. Selective separation of bromoalkane isomers
10. Reversibility and recycling
11. References

1. Materials

All bromoalkanes including 1-bromobutane (1-BBU, ≥ 98%), 2-bromobutane (2-BBU, ≥ 98%), 1-bromopentane (1-BPE, ≥ 98%), 3-bromopentane (3-BPE, ≥ 98%), 1-bromohexane (1-BHE, ≥ 99%), and 2-bromohexane (2-BHE, ≥ 70%, contains 3-bromohexane, stabilized with Copper chip) were purchased from Energy Chemical Company without further purification. 3,5-dinitrobenzonitrile (DNB, ≥ 98%) was purchased from Meryer Chemical Company without further purification. EtP5 was prepared according to literature procedures (*1*). All bromoalkanes isomer mixtures are volume ratio, such as 1:1 (v/v), 1:99 (v/v), 99:1 (v/v).

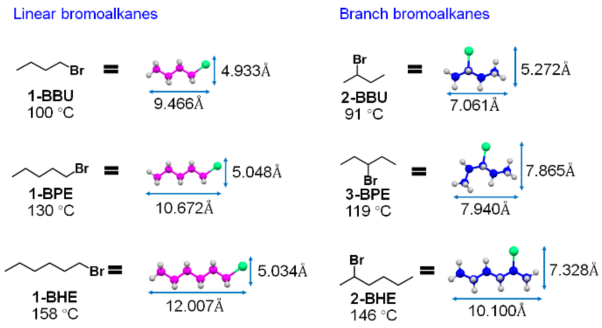

Figure S1. Chemical structures and boiling point of bromoalkanes isomer.

1.1 Single crystals growth

E-D-a red cocrystals were obtained by slow evaporation a CH_2Cl_2 (2 mL) solution of EtP5 (30mg) and DNB (14mg) after 3 days. 2-BBU@E-D red cocrystals were grown by slow evaporation a 2-BBU and CH_2Cl_2 mixed solutions (2 mL) of EtP5 (30 mg) and DNB (14 mg). After 5 days, crystals suitable for X-ray structural determination were obtained. EtP5 (5 mg) was dissolved in 1-BBU (1 ml) and PE was slowly diffused to obtain colorless block crystals 1-BBU@EtP5 suitable for X-ray diffraction.

1.2 Vaporhromic experiments

An open 4 mL vial containing 20 mg of E-D cocrystal or EtP5 was placed in a sealed 20 mL vial containing 1 mL of each bromoalkane solution (*2*). E-D or EtP5 powders were exposed under saturated vapor pressure in the closed vessel at room temperature. Obvious color changes of E-D powders were observed over time. Obvious color changes of EtP5 powders were not observed over time.

2. Methods

2.1 Solution NMR

^{1}H NMR, ^{13}C NMR, and 2D NOESY spectroscopy were measured in $CDCl_3$ using a Bruker Avance III 400 MHz nuclear magnetic resonance spectrometer.

2.2 Solid-state NMR

^{13}C CPMAS NMR was performed at a static magnetic field of 9.4 T with a Bruker Avance III wide-bore NMR spectrometer with a Bruker 4 mm probe at a ^{1}H NMR frequency of 600 MHz.

2.3 Single crystal X-ray diffraction

Single crystal X-ray diffraction data of 1-BBU@EtP5 were collected on a Bruker Smart APXE II CCD diffractometer using Mo-Kα radiation (λ = 0.71073 Å). Single crystal X-ray diffraction data of E-D-a and 2-BBU@E-D were determined by Bruker D8 Venture diffractometer (Ga-Kα radiation, λ = 1.34139 Å).

2.4 Solid-state UV-Vis spectra

Solid-state UV-Vis spectra were recorded on a SHIMADZU UV-2600 spectrophotometer.

2.5 Thermogravimetric Analysis

DSC-TG was carried out using a Mettler Toledo-TGA/DSC3+ instrument and the samples were heated under air gas at a rate of 10 °C / min.

2.6 Powder X-ray diffraction (PXRD)

Powder X-ray diffraction patterns were obtained at ambient (30 °C) temperature by a Bruker D2 with Cu-Kα radiation (λ = 1.5406Å) at 30 kV and 10 mA. The samples were placed onto a circular sample holder and leveled with a glass slide. The sample was scanned within the scan range of 2θ from 5° to 50° continuous scan with a step size of 0.02° and a scan speed of 0.2 s per step.

2.7 Fourier Transform Infrared Spectroscopy (FT-IR)

The FT-IR spectra have been measured on a Perkin Elmer 480 FT-IR spectrophotometer (KBr pellet).

2.8 Raman Spectroscopy

The Raman spectroscopy has been measured on a Horiba scientific-LabRAM HR evolution.

2.9 Optical images

Optical images were taken with a Canon 70D camera.

2.10 Electrochemical Characterization

The Mott–Schottky spots were carried out with CHI 660E electrochemical workstation. A 10 mg powder sample was weighed and dispersed in 1 mL of ultrapure water, then an appropriate amount of conductive binder was added and sonicated for 30 min to form a homogeneous suspension, then 150 μL of the suspension was added dropwise on the FTO glass and dried at room temperature. The test was performed with a three-electrode system, with FTO as the working electrode, silver/silver chloride as the reference electrode, and platinum sheet as the counter electrode. 0.2 M Na_2SO_4 aqueous sodium was used as the electrolyte, and the photoelectric test was performed under a nitrogen atmosphere. The conductive binder is selected from polyvinyl alcohol or sodium carboxymethyl cellulose, and the test frequency is selected from 500, 1000, and 1500 Hz.

3. Choosing a suitable pillararene

3.1 EtP5 selective adsorption of BBU

An open 4 mL vial containing 20 mg of EtP5 was placed in a sealed 20 mL vial containing 1 mL of each bromoalkane solution. EtP5 powders were exposed under saturated vapor pressure in the closed vessel at room temperature. Obvious color changes were not observed over time. All bromoalkanes isomer mixtures are volume ratio, such as 1:1 (v/v), 1:99 (v/v), 99:1 (v/v). Before measurement, the powders were heated at 40 °C to remove the surface-physically adsorbed vapor.

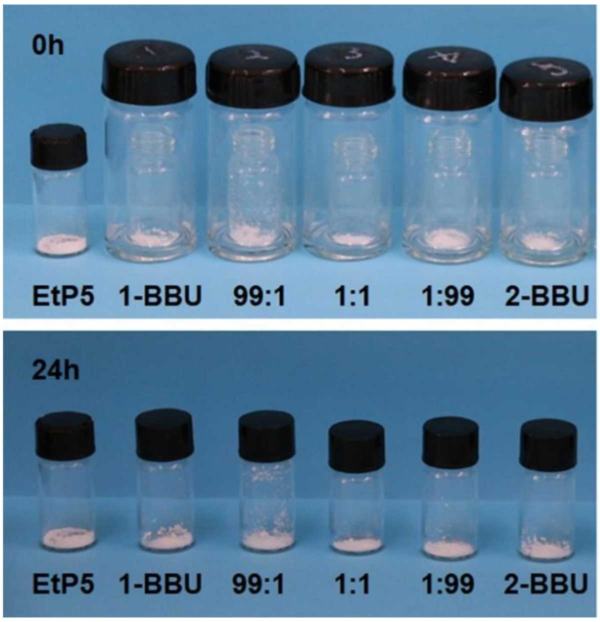

Figure S2. The color of EtP5 no changes in vapor-phase adsorption experiments at 0 h and 24 h.

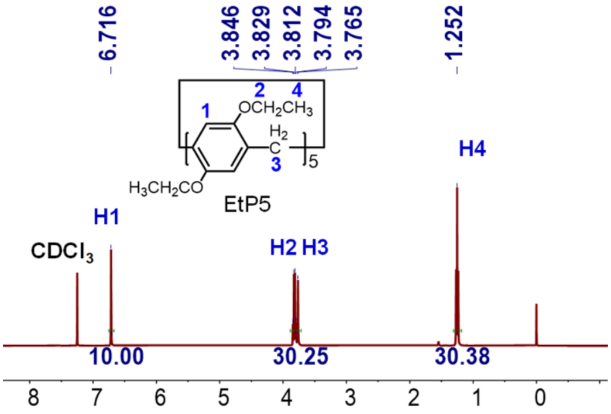

Figure S3. ^{1}H NMR spectrum (400 MHz, 298 K, $CDCl_3$) of EtP5.

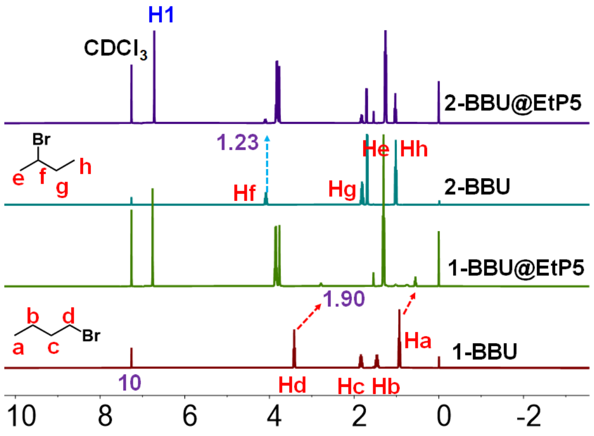

Figure S4. ^{1}H NMR spectrum (400 MHz, 298 K, $CDCl_3$) of 1-BBU, 1-BBU@EtP5 (EtP5 after adsorption of 1-BBU), 2-BBU, and 2-BBU@EtP5 (EtP5 after adsorption of 2-BBU). ^{1}H NMR experiments were performed by dissolving the EtP5 powders after the vapor adsorption experiment in $CDCl_3$.

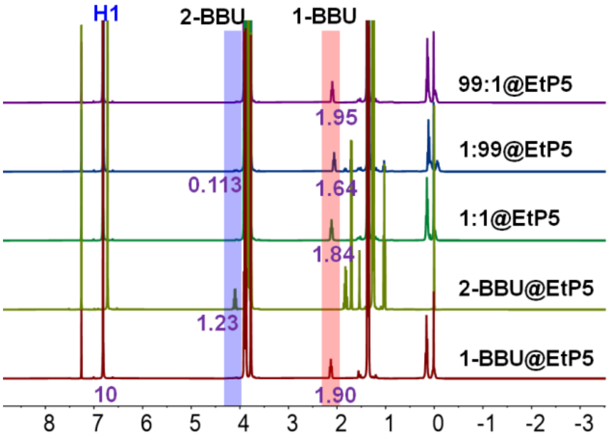

Figure S5. ^{1}H NMR spectrum (400 MHz, 298 K, $CDCl_3$) of 1-BBU@EtP5, 2-BBU@EtP5, 1:1@EtP5 (EtP5 after adsorption of 1-BBU:2-BBU = 1:1 (v/v) mixed vapor), 1:99@EtP5 (EtP5 after adsorption of 1-BBU:2-BBU = 1:99 (v/v) mixed vapor), and 99:1@EtP5 (EtP5 after adsorption of 1-BBU:2-BBU = 99:1 (v/v) mixed vapor) for 24 h. ^{1}H NMR experiments were performed by dissolving the EtP5 powders after the vapor adsorption experiment in $CDCl_3$.

Table S1. Selectivity and adsorption capacity of 1-BBU.

Vapor-phase	1-BBU	2-BBU	1:99	1:1	99:1
Selectivity of 1-BBU	100%	0%	87.85%	100%	100%
Adsorption capacity (n (EtP5: 1-BBU))	1:0.95	-	1:0.82	1:0.92	1:0.975
Adsorption capacity (n (EtP5: 2-BBU))	-	1:1.23	1:0.113	1:0	1:0

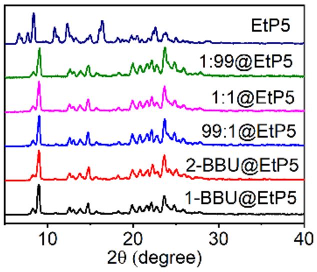

Figure S6. The PXRD patterns of 1-BBU@EtP5, 2-BBU@EtP5, 99:1@EtP5, 1:1@EtP5, 1:99@EtP5, and EtP5.

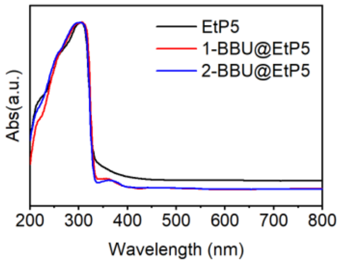

Figure S7. Normalized solid-state UV-Vis spectra of EtP5, 1-BBU@EtP5, and 2-BBU@EtP5.

3.2 Guest exchange adsorption experiments of 1-BBU@EtP5

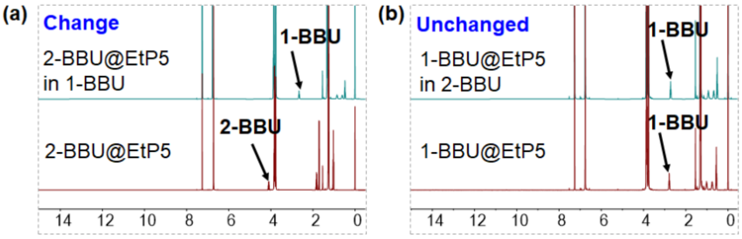

Figure S8. Guest exchange of 1-BBU@EtP5 and 2-BBU@EtP5. ^{1}H NMR spectra of (a) 2-BBU@EtP5 and guest exchange with 1-BBU after 8 h; (b) 1-BBU@EtP5 and guest exchange with 2-BBU after 8 h.

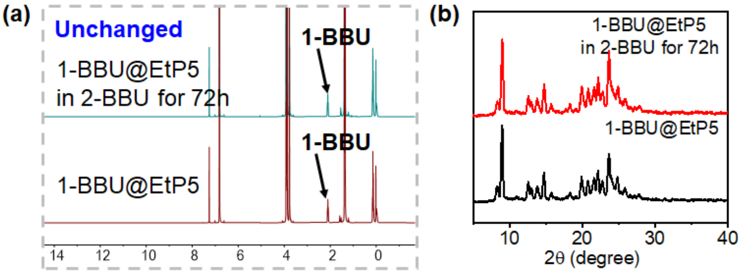

Figure S9. Guest exchange of 1-BBU@EtP5. (a) ^{1}H NMR spectrum and (b) PXRD patterns of 1-BBU@EtP5 adsorption of 2-BBU for 72 h, 1-BBU@EtP5.

3.3 Desorption experiments of BBU

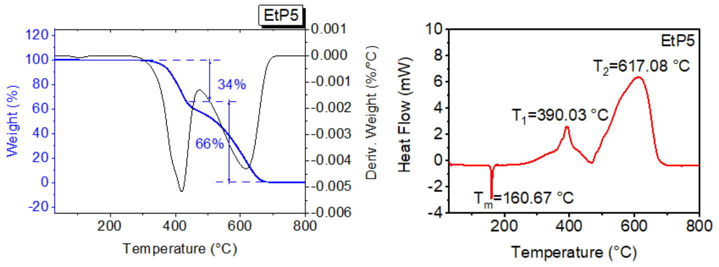

Figure S10. DSC-TG of EtP5 (air atmosphere).

EtP5 loses weight in the air in two stages, the first stage is the consumption of its O atoms, and the second stage is the consumption of air, corresponding to 38 %, and 62 %, respectively. The melting point of EtP5 is 160.67 °C.

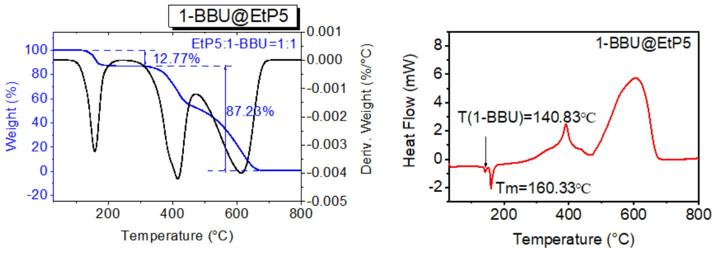

Figure S11. DSC-TG of 1-BBU@EtP5 (air atmosphere).

The EtP5:1-BBU=1:1. The desorption temperature of 1-BBU is 140.83 °C. The melting point of EtP5 is 160.33 °C.

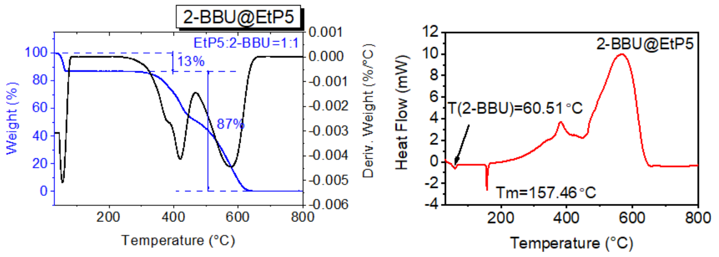

Figure S12. DSC-TG of 2-BBU@EtP5 (air atmosphere).

The EtP5:2-BBU=1:1. The desorption temperature of 2-BBU is 60.51 °C. The melting point of EtP5 is 157.46 °C.

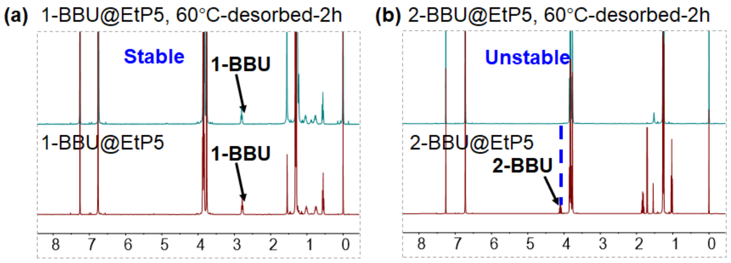

Figure S13. Guest desorption properties of 1-BBU@EtP5 and 2-BBU@EtP5. (a) 1-BBU@EtP5 and 1-BBU@EtP5 after desorption for 2 h at 60 °C; (b) 2-BBU@EtP5 and 2-BBU@EtP5 after desorption for 2 h at 60 °C.

4. Choosing a matching acceptor

We calculated the intermolecular binding energy and electrostatic potential, using the DFT-D3 method (DFT including the D3 version of Grimme's dispersion) at the B3LYP/6-31G level to predict their pore-outside intermolecular interactions.

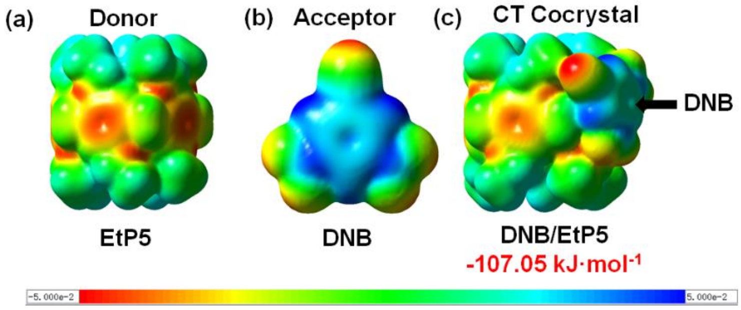

Figure S14. Distributions of the electrostatic potential mapped onto the electron density surfaces of (a) EtP5, (b) DNB, and (c) E-D Cocrystal. The binding energy of DNB/EtP5 is -107.05 kJ·mol^{-1}.

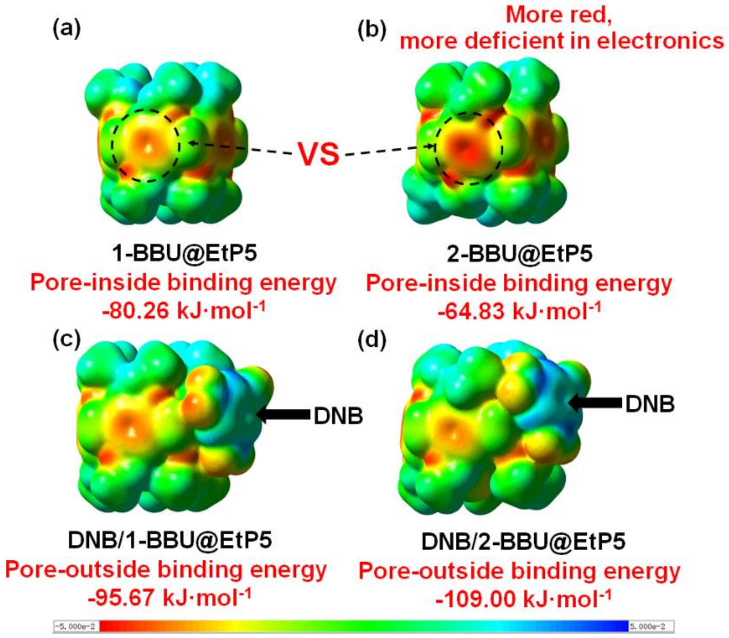

Figure S15. Distributions of the electrostatic potential mapped onto the electron density surfaces of (a) 1-BBU@EtP5 (the binding energy is -80.26 kJ·mol^{-1}), (b) 2-BBU@EtP5 (the binding energy is -64.83 kJ·mol^{-1}), (c) DNB/1-BBU@EtP5 (the binding energy is -95.67 kJ·mol^{-1}), and (d) DNB/2-BBU@EtP5 (the binding energy is -109.00 kJ·mol^{-1}).

5. Synthesis and characterization of cocrystal

5.1 Characterization of E-D-a Cocrystal

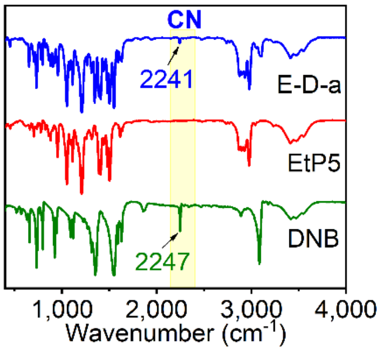

Figure S16. The FT-IR of E-D-a cocrystal, EtP5, and DNB.

After the formation of cocrystal between EtP5 and DNB, the CN group of DNB was shifted from 2247 cm^{-1} to 2241 cm^{-1}, suggesting charge transfer interaction between EtP5 and DNB.

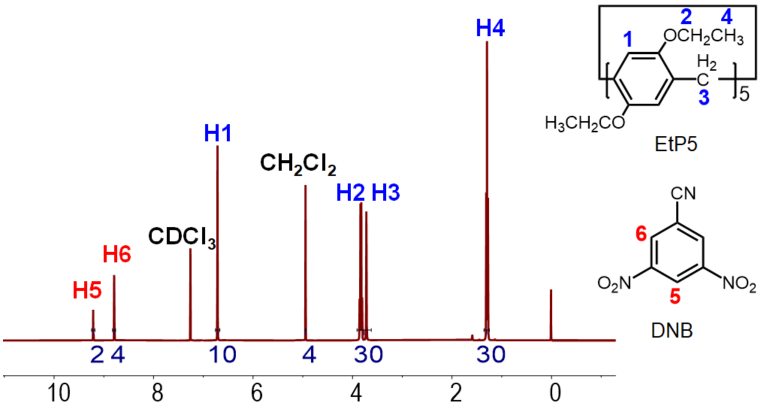

Figure S17. ^{1}H NMR spectrum (400 MHz, 298 K, $CDCl_3$) of E-D-a cocrystal.

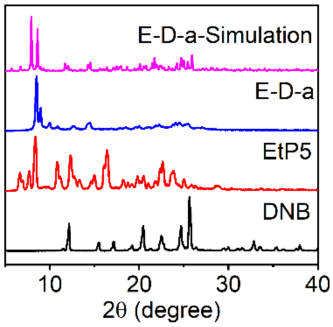

Figure S18. The PXRD patterns: DNB, EtP5, E-D-a cocrystal, and simulated from the single crystal structure of E-D-a.

5.2 Characterization of E-D Cocrystal

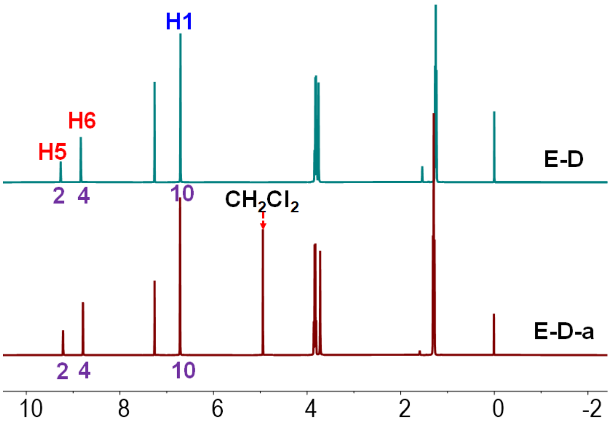

Figure S19. ^{1}H NMR spectrum (400 MHz, 298 K, $CDCl_3$) of E-D-a and E-D cocrystal.

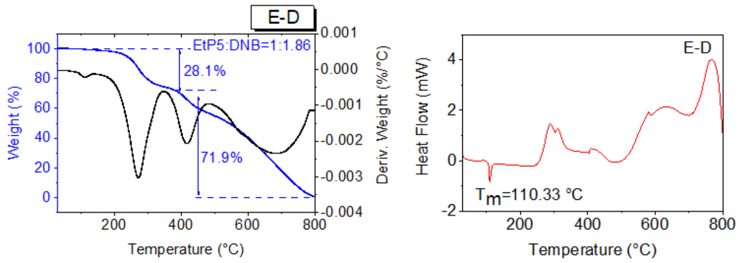

Figure S20. DSC-TG of E-D (air atmosphere).

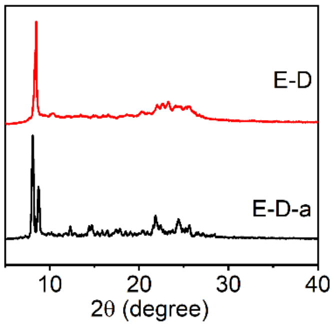

Figure S21. The PXRD patterns of E-D and E-D-a.

6. E-D cocrystal adsorption single component bromoalkane

6.1 Characterization of E-D cocrystal after adsorption BBU

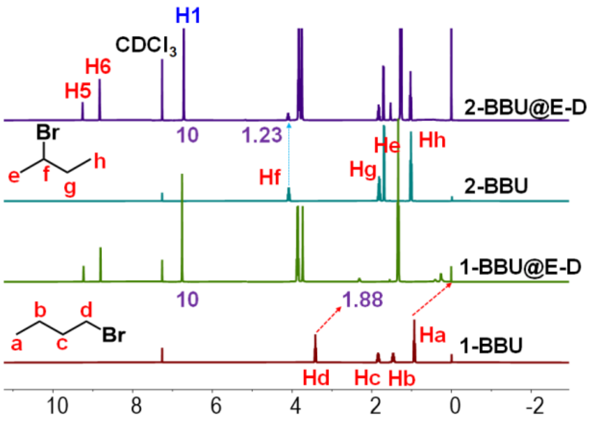

Figure S22. ^{1}H NMR spectrum (400 MHz, 298 K, $CDCl_3$) of 1-BBU, 1-BBU@E-D (the uptake amount was calculated as 0.94 mol 1-BBU/mol E-D), 2-BBU, and 2-BBU@E-D (the uptake amount was calculated as 1.23 mol 2-BBU/mol E-D).

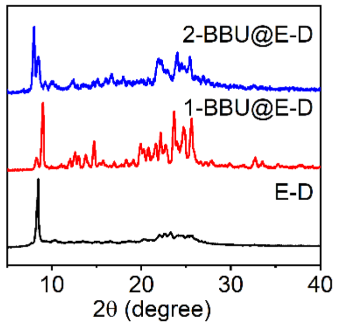

Figure S23. The PXRD patterns of E-D cocrystal, 1-BBU@E-D, and 2-BBU@E-D.

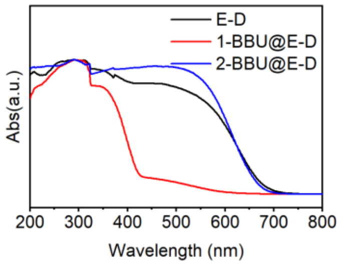

Figure S24. Normalized solid-state UV-Vis spectra of E-D cocrystal, 1-BBU@E-D, and 2-BBU@E-D.

6.2 Time-dependent solid-vapor adsorption experiment

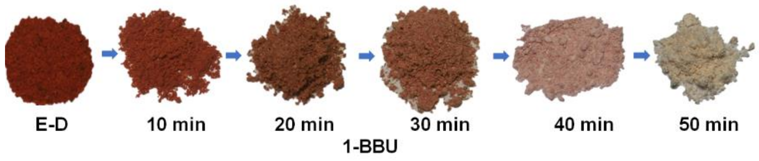

Figure S25. Color change of E-D cocrystal powder in 1-BBU vapor with time.

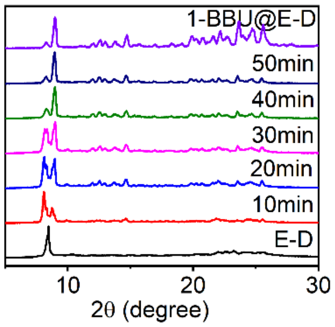

Figure S26. The PXRD patterns of E-D, E-D after adsorption 1-BBU vapor for 10 min, 20 min, 30 min, 40 min, 50 min, and 1-BBU@E-D.

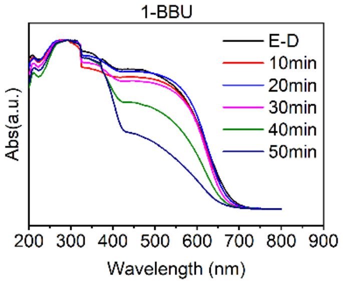

Figure S27. Normalized solid-state UV-Vis spectra of E-D, and E-D after adsorption 1-BBU vapor for 10 min, 20 min, 30 min, 40 min, and 50 min.

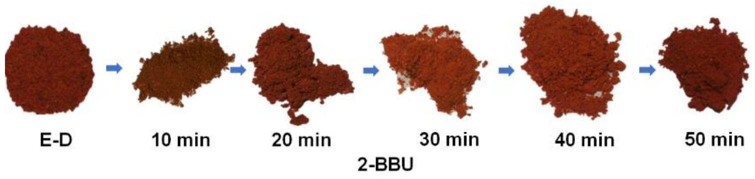

Figure S28. Color change of E-D cocrystal powder in 2-BBU vapor with time.

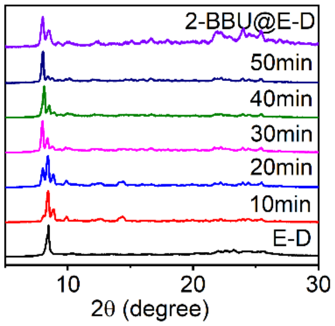

Figure S29. The PXRD patterns of E-D, E-D after adsorption 2-BBU vapor for 10 min, 20 min, 30 min, 40 min, 50 min, and 2-BBU@E-D.

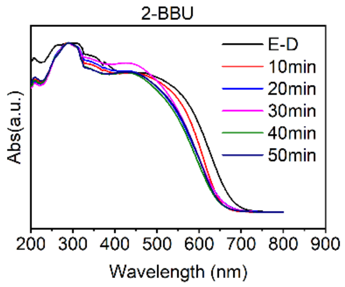

Figure S30. Normalized solid-state UV-Vis spectra of E-D, and E-D after adsorption 2-BBU vapor for 10 min, 20 min, 30 min, 40 min, and 50 min.

6.3 Characterization of E-D cocrystal after adsorption BPE

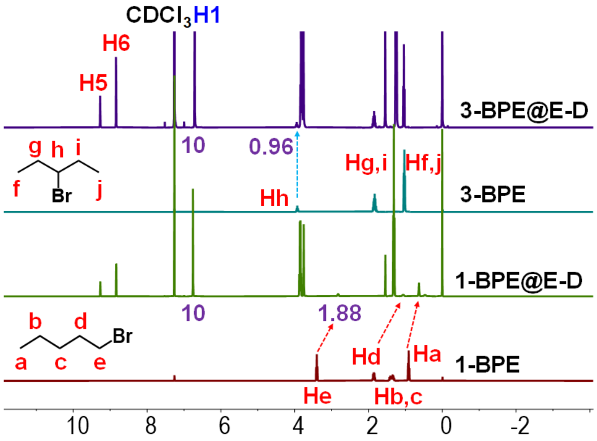

Figure S31. ^{1}H NMR spectrum (400 MHz, 298 K, $CDCl_3$) of 1-BPE, 1-BPE@E-D (the uptake amount was calculated as 0.94 mol 1-BPE/mol E-D), 3-BPE, and 3-BPE@E-D (the uptake amount was calculated as 0.96 mol 1-BPE/mol E-D).

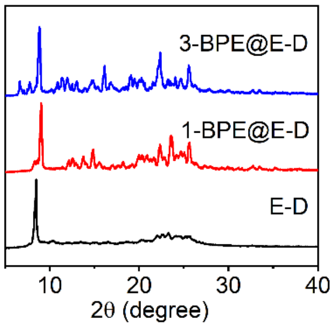

Figure S32. The PXRD patterns of 3-BPE@E-D, 1-BPE@E-D, and E-D cocrystal.

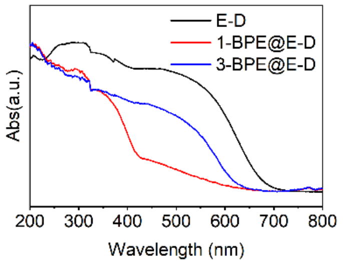

Figure S33. Normalized solid-state UV-Vis spectra of 3-BPE@E-D, 1-BPE@E-D, and E-D cocrystal.

6.4 Characterization of E-D cocrystal after adsorption BHE

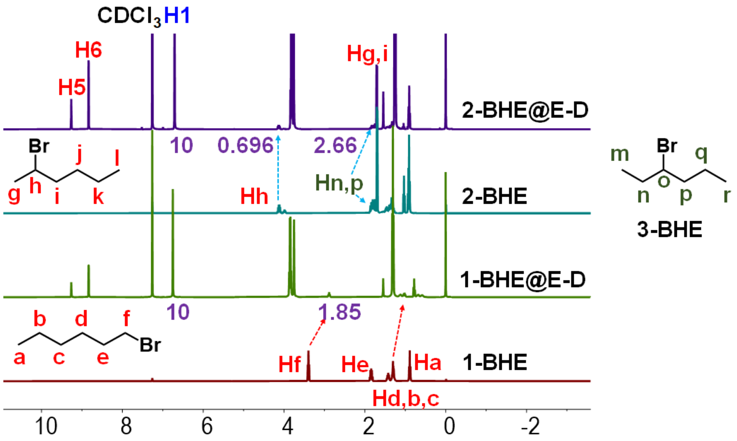

Figure S34. ^{1}H NMR spectrum (400 MHz, 298 K, $CDCl_3$) of 1-BHE, 1-BHE@E-D (the uptake amount was calculated as 0.925 mol 1-BHE/mol E-D), 2-BHE, and 2-BHE@E-D (the uptake amount was calculated as 0.696 mol 2-BHE/mol E-D and 0.665 mol 3-BHE/mol E-D).

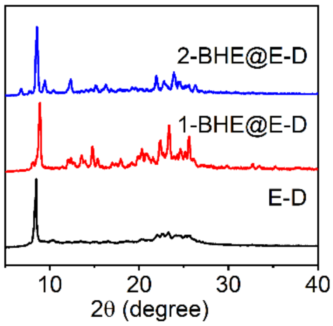

Figure S35. The PXRD patterns of E-D cocrystal, 1-BHE@E-D, and 2-BHE@E-D.

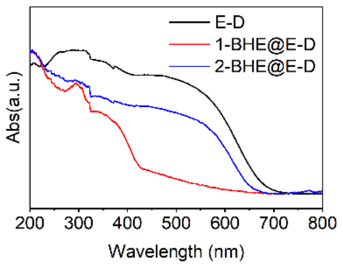

Figure S36. Normalized solid-state UV-Vis spectra of E-D cocrystal, 1-BHE@E-D, and 2-BHE@E-D.

7. Investigation of intermolecular interactions

7.1 Pore-outside CT interactions

(1) The solid-state cross-polarization magic-angle spinning (CP/MAS) ^{13}C NMR spectrum

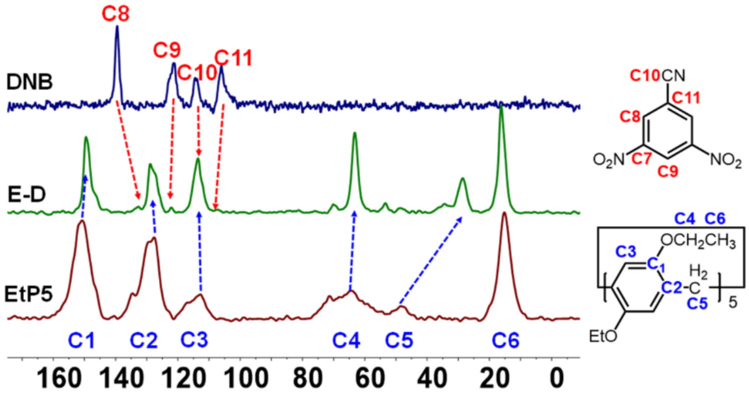

Figure S37. CP/MAS ^{13}C NMR spectrum (600MHz) of EtP5, DNB, and E-D cocrystal.

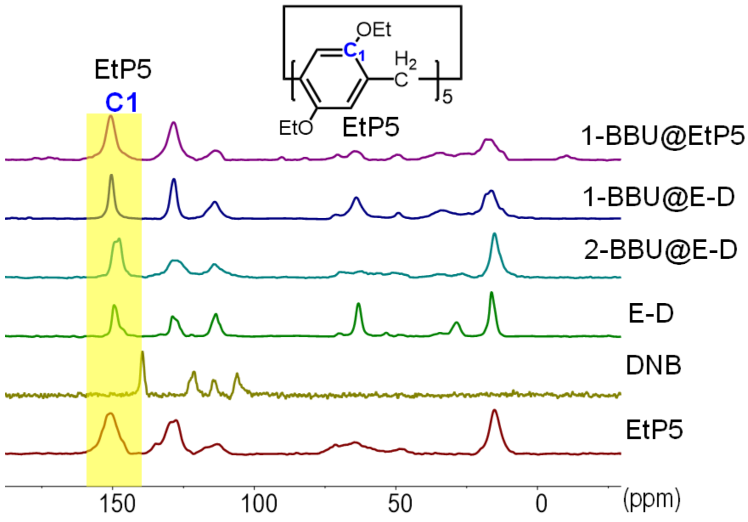

Figure S38. CP/MAS ^{13}C NMR spectrum (600MHz) of EtP5, DNB, E-D cocrystal, 2-BBU@E-D, 1-BBU@E-D, and 1-BBU@EtP5.

(2) FT-IR

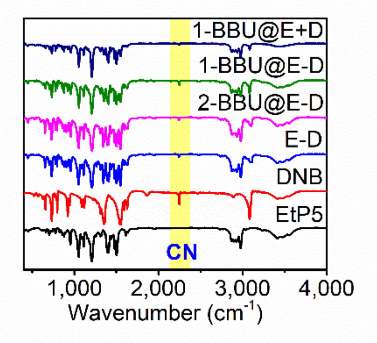

Figure S39. The FT-IR of EtP5, DNB, E-D cocrystal, 1-BBU@E-D, 2-BBU@E-D, and 1-BBU@E+D.

2-BBU@E-D was almost unchanged and the corresponding characteristic peaks were not chemically shifted. The CN group was shifted from 2241 cm^{-1} to 2247 cm^{-1} after cocrystal adsorption of 1-BBU, similar to DNB and 1-BBU@E+D.

(3) PXRD

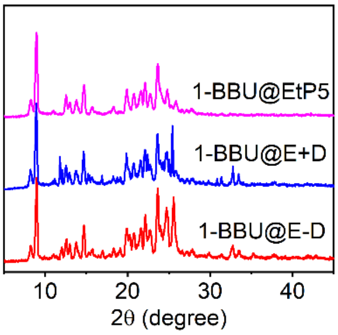

Figure S40. The PXRD patterns of 1-BBU@E-D, 1-BBU@E+D, and 1-BBU@EtP5.

(4) Raman

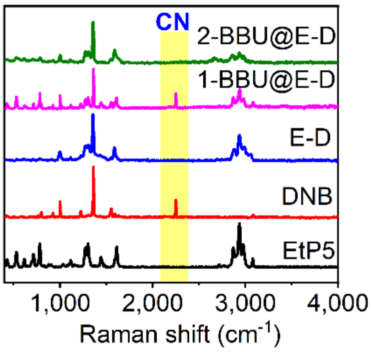

Figure S41. The Raman of EtP5, DNB, E-D cocrystal, 1-BBU@E-D, and 2-BBU@E-D.

(5) Calculated band gap from electrochemical properties

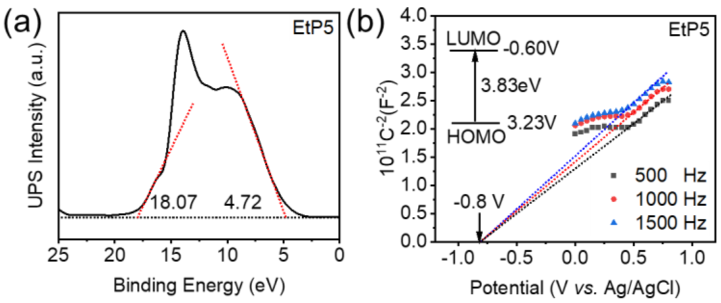

Figure S42. (a) Ultraviolet photoelectron spectroscopy (UPS) spectrum of EtP5. (b) Mott-Schottky plots for EtP5 in 0.2M Na_2SO_4 aqueous solution. Insets are the energy diagrams of the HOMO and LUMO levels of the EtP5.

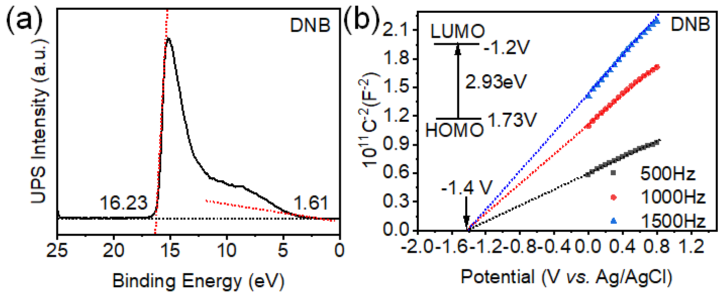

Figure S43. (a) Ultraviolet photoelectron spectroscopy (UPS) spectrum of DNB. (b) Mott-Schottky plots for DNB in 0.2M Na_2SO_4 aqueous solution. Insets are the energy diagrams of the HOMO and LUMO levels of the DNB.

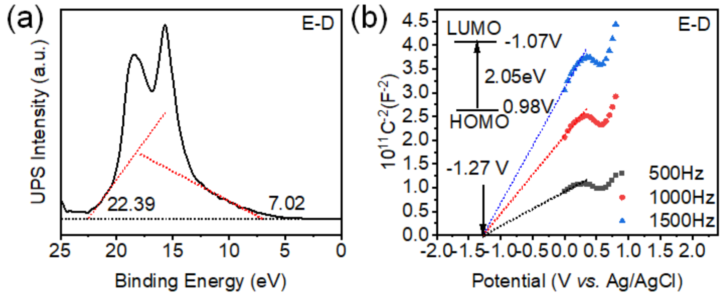

Figure S44. (a) Ultraviolet photoelectron spectroscopy (UPS) spectrum of E-D cocrystal. (b) Mott-Schottky plots for E-D cocrystal in 0.2M Na_2SO_4 aqueous solution. Insets are the energy diagrams of the HOMO and LUMO levels of the E-D cocrystal.

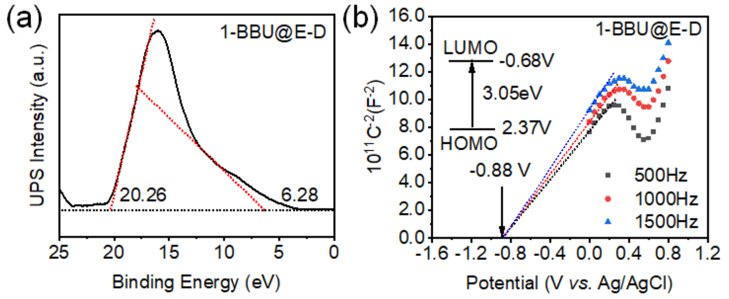

Figure S45. (a) Ultraviolet photoelectron spectroscopy (UPS) spectrum of 1-BBU@E-D. (b) Mott-Schottky plots for 1-BBU@E-D in 0.2M Na_2SO_4 aqueous solution. Insets are the energy diagrams of the HOMO and LUMO levels of the 1-BBU@E-D.

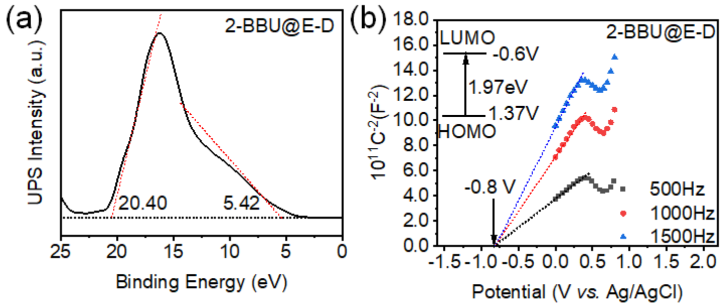

Figure S46. (a) Ultraviolet photoelectron spectroscopy (UPS) spectrum of 2-BBU@E-D. (b) Mott-Schottky plots for 2-BBU@E-D in 0.2M Na_2SO_4 aqueous solution. Insets are the energy diagrams of the HOMO and LUMO levels of the 2-BBU@E-D.

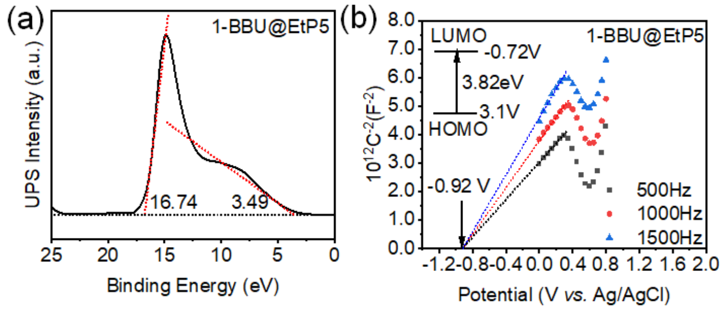

Figure S47. (a) Ultraviolet photoelectron spectroscopy (UPS) spectrum of 1-BBU@EtP5. (b) Mott-Schottky plots for 1-BBU@EtP5 in 0.2M Na_2SO_4 aqueous solution. Insets are the energy diagrams of the HOMO and LUMO levels of the 1-BBU@EtP5.

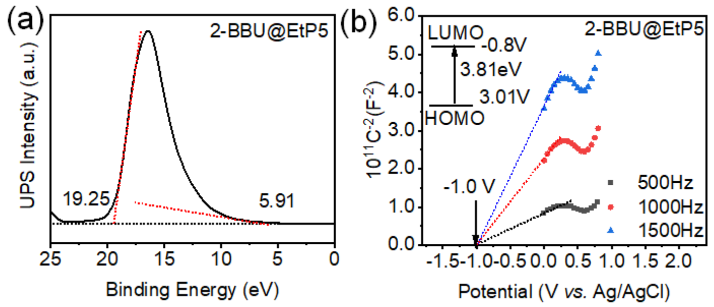

Figure S48. (a) Ultraviolet photoelectron spectroscopy (UPS) spectrum of 2-BBU@EtP5. (b) Mott-Schottky plots for 2-BBU@EtP5 in 0.2M Na_2SO_4 aqueous solution. Insets are the energy diagrams of the HOMO and LUMO levels of the 2-BBU@EtP5.

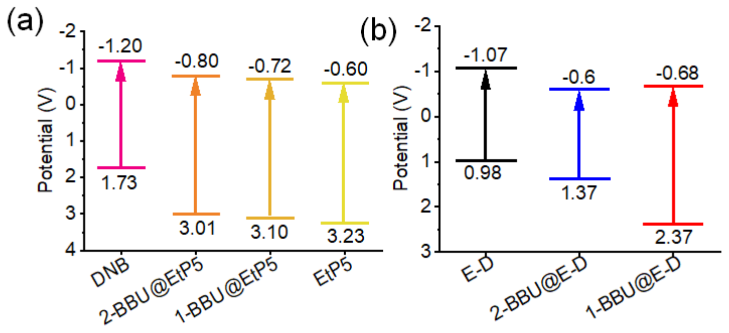

Figure S49. HOMO-LUMO position images of (a) DNB, 2-BBU@EtP5, 1-BBU@EtP5, EtP5, and (b) E-D cocrystal, 2-BBU@E-D, 1-BBU@E-D.

(6) Band gaps of solid-state UV-Vis experiments

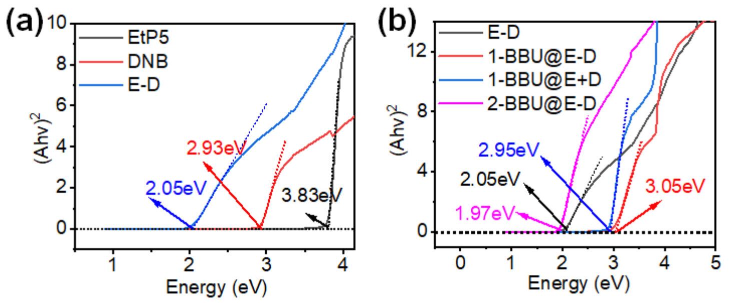

Figure S50. Solid-state UV-Vis spectra band gap of (a) EtP5, DNB, and E-D cocrystal, (b) E-D cocrystal, 1-BBU@E-D, 2-BBU@E-D, and 1-BBU@E+D.

7.2 Pore-inside intermolecular interactions

(1) Guest exchange adsorption experiment of BBU

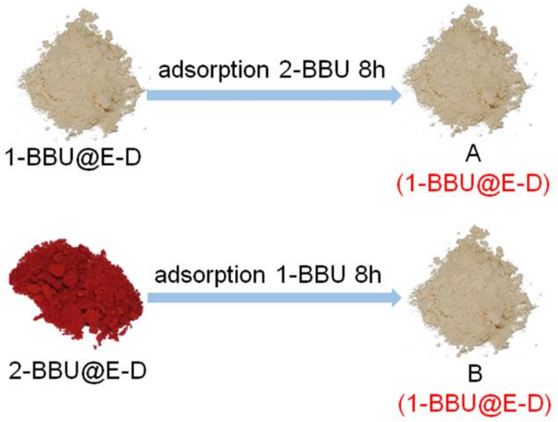

Figure S51. The color changes of guest exchange adsorption experiments: 1-BBU@E-D, A (1-BBU@E-D adsorption in 2-BBU for 8 h); 2-BBU@E-D, B (2-BBU@E-D adsorption in 1-BBU for 8 h).

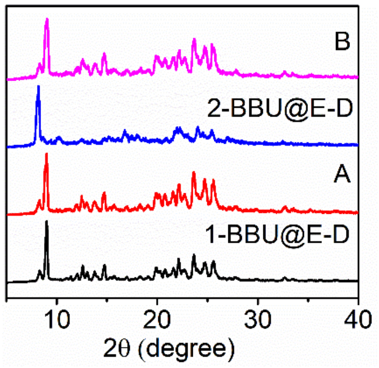

Figure S52. The PXRD patterns of 1-BBU@E-D, A, 2-BBU@E-D, and B.

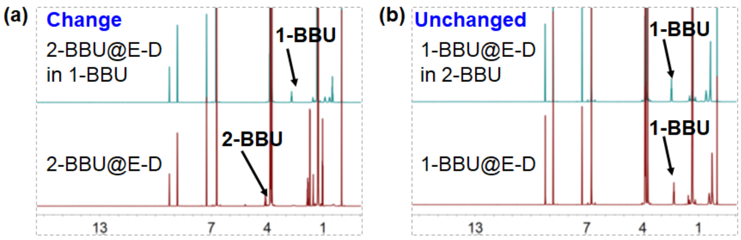

Figure S53. Guest exchange by 1-BBU@E-D and 2-BBU@E-D. ^{1}H NMR spectra of (a) 2-BBU@E-D and guest exchange with 1-BBU after 8 h; (b) 1-BBU@E-D and guest exchange with 2-BBU after 8 h.

(2) DSC-TG

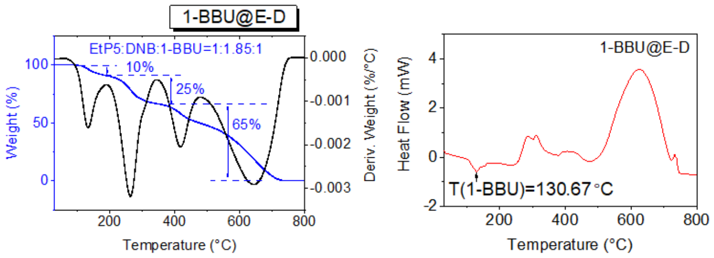

Figure S54. DSC-TG of 1-BBU@E-D (air atmosphere).

The EtP5: DNB: 1-BBU=1:1.85:1. The desorption temperature of 1-BBU is 130.67 °C.

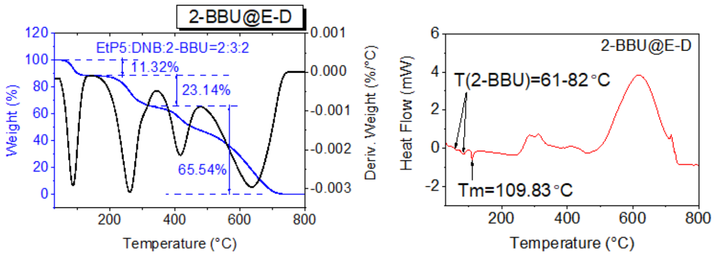

Figure S55. DSC-TG of 2-BBU@E-D (air atmosphere).

The EtP5: DNB: 2-BBU=2:3:2. The desorption temperature of 2-BBU is 61- 82 °C. The melting point of cocrystal is 109.83 °C, similar to E-D.

(3) Guest desorption experiment of BBU

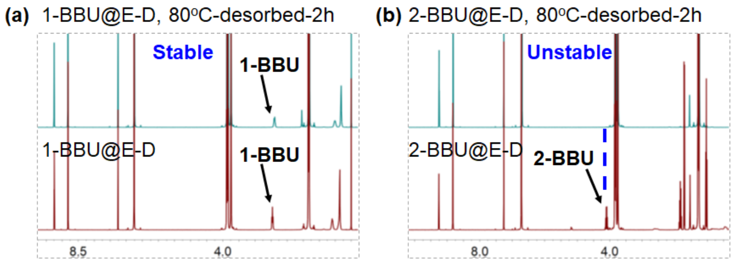

Figure S56. Guest desorption by 1-BBU@E-D and 2-BBU@E-D. ^{1}H NMR spectra of (a) 1-BBU@E-D and 1-BBU@E-D after desorption for 2 h at 80 °C; (b) 2-BBU@E-D and 2-BBU@E-D after desorption for 2 h at 80 °C.

(4) Guest exchange adsorption experiment of 1-BBU@E+D

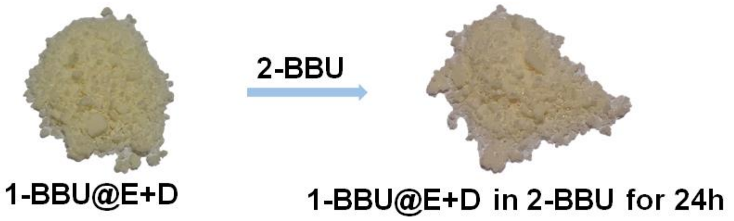

Figure S57. The color changes of 1-BBU@E+D and 1-BBU@E+D adsorption in 2-BBU for 24 h.

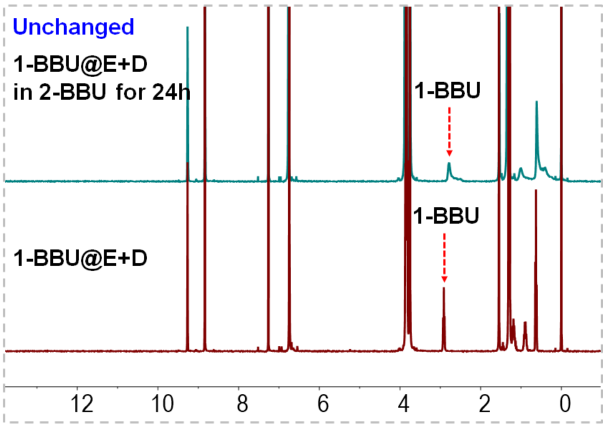

Figure S58. ^{1}H NMR spectrum of 1-BBU@E+D and 1-BBU@E+D adsorption in 2-BBU for 24 h.

(5) The desorption experiments of 1-BBU@E+D

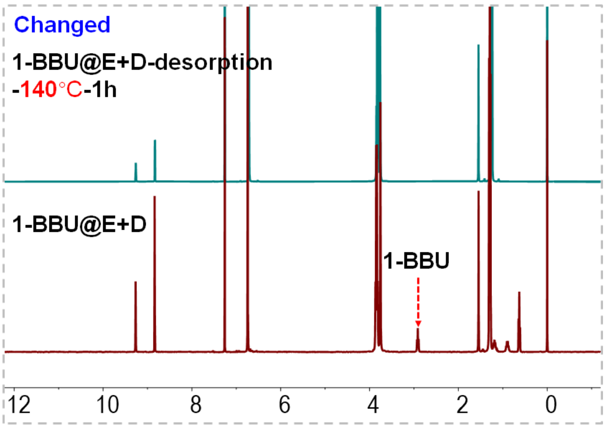

Figure S59. ^{1}H NMR spectrum of the before and after desorption experiments in 1-BBU@E+D.

8. Crystal data and structures

Table S2. Crystal data and structure refinement for cocrystal.

Formula	E-D-a	1-BBU@EtP5	2-BBU@E-D
Crystallization Solvent	CH_2Cl_2	1-BBU	2-BBU
Formula	$C_{71}H_{80}Cl_4N_6O_{18}$	$C_{59}H_{79}BrO_{10}$	$C_{139}H_{167}Br_2N_9O_{32}$
Formula weight	1447.21	1024.10	2635.63
Temperature / K	150	200.0	150.0
Crystal system	monoclinic	orthorhombic	monoclinic
Space group	P 1 21/n 1 (14)	Pbcn	$P2_1/n$
a / Å	14.9776(6)	43.289(6)	24.1436(16)
b / Å	26.3238	15.773(2)	11.7948(8)
c / Å	19.1584	16.194(2)	50.385(3)
α /°	90	90	90
β/ °	96.466(2)	90	95.332(3)
γ/ °	90	90	90
Volume / $Å^3$	7505.5(5)	11057(2)	14286.1(16)
Z	4	8	4
ρ_{calc} g/cm^3	1.281	1.230	1.225
μ mm^{-1}	1.314	0.799	0.888
Crystal size / mm^3	-	0.13 × 0.12 × 0.1	-
Radiation	Ga-Kα (λ= 1.34139)	Mo-Kα (λ= 0.71073)	Ga-Kα (λ = 1.34139)
F (000)	3032.0	4352.0	5560.0
2θ range for data collection / °	5.842 to 109.906	2.748 to 54.292	5.352 to 106.044
Index range	-18≤ h ≤17, -32 ≤ k ≤32, -23 ≤ l ≤23	-53 ≤ h ≤ 55, -20 ≤ k ≤ 20, -16 ≤ l ≤ 20	-28 ≤ h ≤ 28, -12 ≤ k ≤ 14, -59 ≤ l ≤ 58
Reflections collected	106323	69787	176540

Independent reflections, R_{int}	14266, 0.0345	12116, 0.0535	25109, 0.0422
Goodness-of-fit on F^2	1.035	1.042	1.032
Final R_1 indexes [I>=2σ(I)]	0.0617	0.0597	0.0672
Final R_1 indexes [all data]	0.0649	0.0794	0.0814
Final wR (F_2) indexes [all data]	0.1892	0.1796	0.2012
Largest diff. peak/hole / e $Å^{-3}$	0.91/-1.67	0.48/-0.39	1.35/-0.95
CCDC number	2191516	2191518	2191528

8.1 Crystal structures of E-D-a

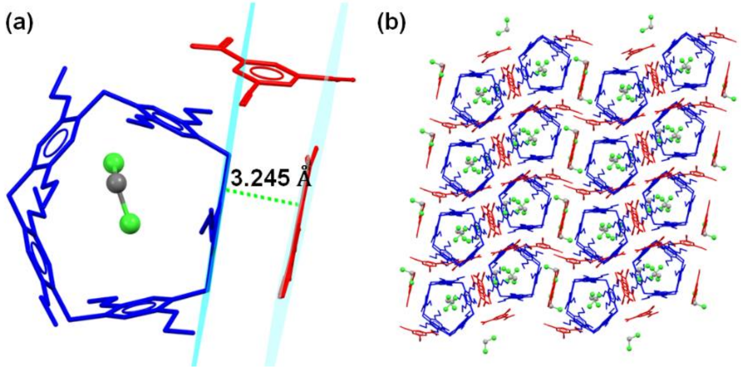

Figure S60. The single crystal structure of E-D-a cocrystal. (a) The pore-outside intermolecular interactions, and (b) the packing view of E-D-a cocrystal. Some protons were omitted for clarity. Color codes: EtP5: blue; DNB, red; C of CH_2Cl_2, gray; Cl, green.

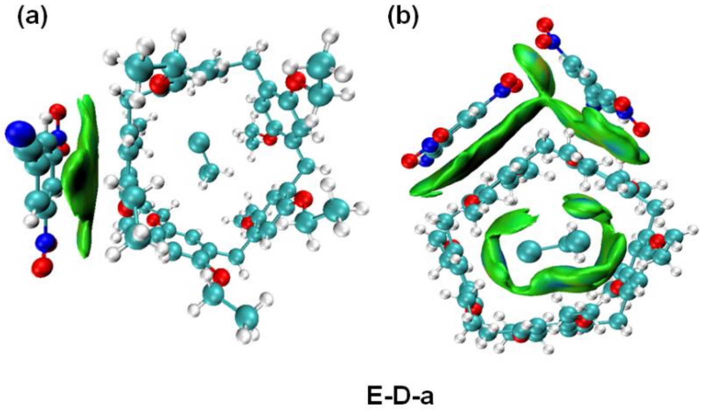

Figure S61. The host-guest interaction of E-D-a cocrystal.

8.2 Crystal structures of 2-BBU@E-D

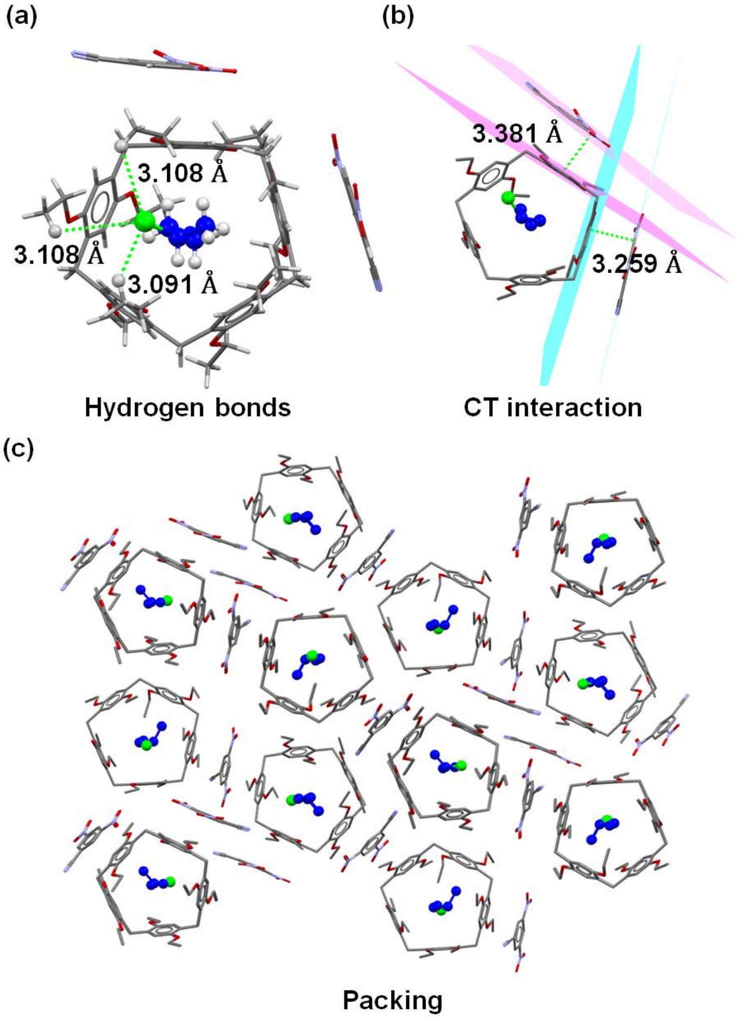

Figure S62. The single crystal structure of 2-BBU@E-D cocrystal. (a) The pore-inside intermolecular interactions, (b) the pore-outside intermolecular interactions, and (c) the packing view of 2-BBU@E-D. Some protons were omitted for clarity. Color codes: Br, green; C of 2-BBU, blue; C of EtP5 and DNB, grey; O of EtP5 and DNB, red; H of EtP5, light grey; N of DNB, navy blue.

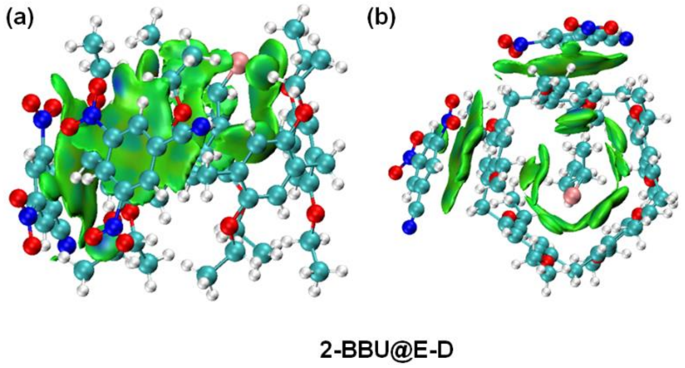

Figure S63. The host-guest interaction of 2-BBU@ E-D.

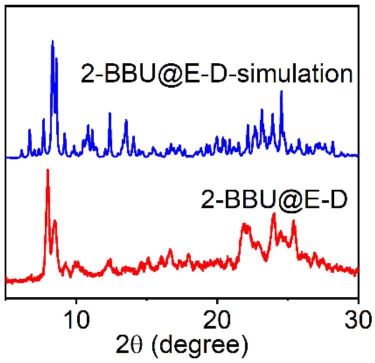

Figure S64. The PXRD patterns: 2-BBU@E-D and simulated from the single crystal structure of 2-BBU@E-D cocrystal.

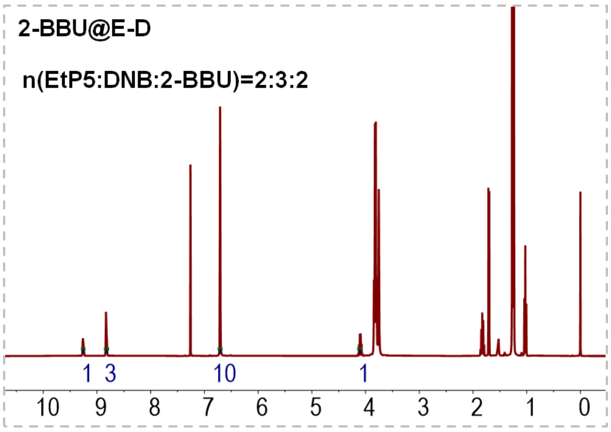

Figure S65. ^{1}H NMR spectrum of 2-BBU@E-D cocrystal.

8.3 Crystal structures of 1-BBU@EtP5

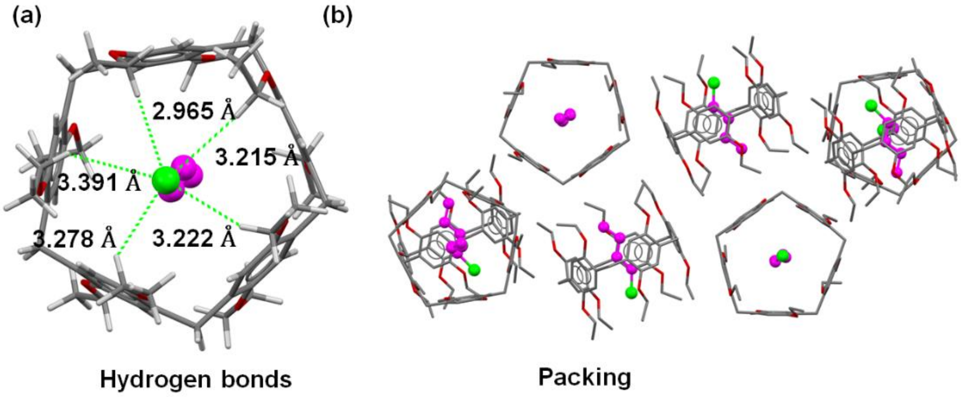

Figure S66. The single crystal structure of 1-BBU@EtP5 cocrystal. (a) The pore-inside intermolecular interactions, and (b) the packing view of 1-BBU@EtP5. Some protons were omitted for clarity. Color codes: Br, green; C of 1-BBU, magenta. C of EtP5, grey; O of EtP5, red; H of EtP5, light grey.

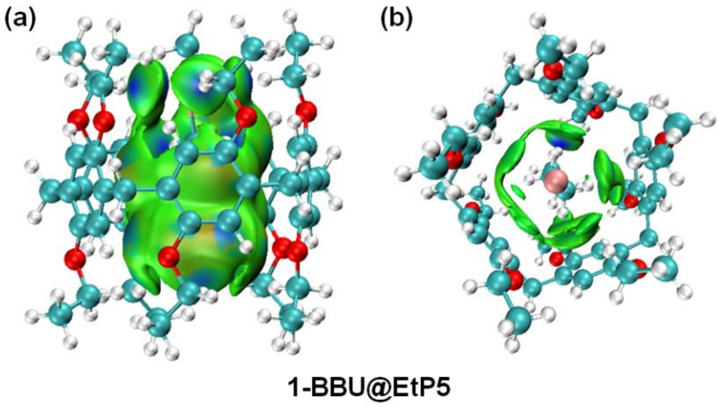

Figure S67. The host-guest interaction of 1-BBU@EtP5.

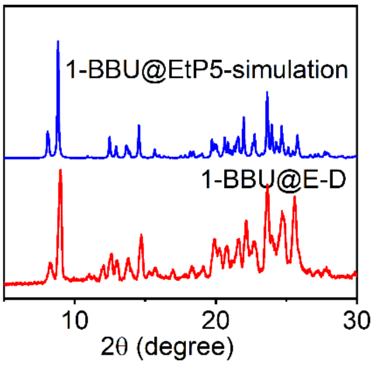

Figure S68. The PXRD patterns: 1-BBU@EtP5 and simulated from the single crystal structure of 1-BBU@EtP5 cocrystal.

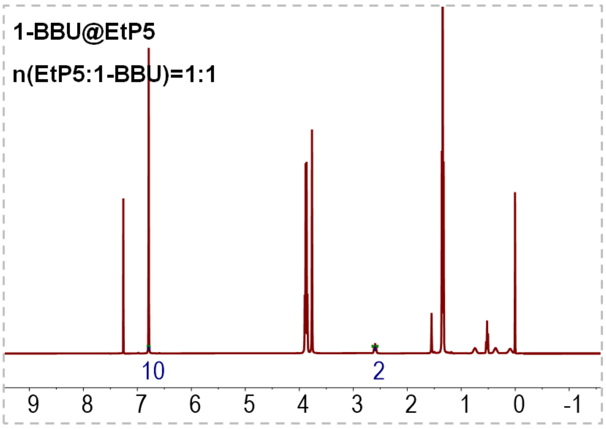

Figure S69. ^{1}H NMR spectrum of 1-BBU@EtP5 cocrystal.

9. Selective separation of bromoalkane isomers

9.1 Time-dependent solid-vapor adsorption experiment of v(1-BBU:2-BBU) = 1:1 mixed vapor for E-D cocrystal

An open 4 mL vial containing 20 mg of E-D powders was placed in a sealed 20 mL vial containing 1 mL of each bromoalkane solution. E-D powders were exposed under saturated vapor pressure in the closed vessel at room temperature. All bromoalkane isomers mixtures are volume ratio 1-BBU:2-BBU 1:1 (v/v). Before measurement, the powders were heated at 40 °C to remove the surface-physically adsorbed vapor.

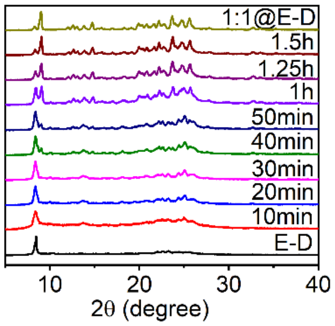

Figure S70. The PXRD patterns of E-D cocrystal, E-D after adsorption in v(1-BBU:2-BBU) = 1:1 mixed vapor for 10 min, 20 min, 30 min, 40 min, 50 min, 1 h, 1.25 h, 1.5 h, and 1:1@E-D.

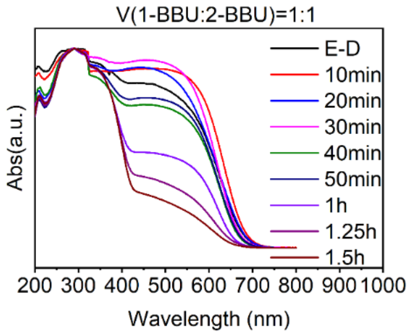

Figure S71. Normalized solid-state UV-Vis spectra of E-D cocrystal, E-D after adsorption in v(1-BBU:2-BBU) = 1:1 mixed vapor for 10 min, 20 min, 30 min, 40 min, 50 min, 1 h, 1.25 h, 1.5 h.

9.2 Characterization of E-D cocrystal after adsorption 1-BBU:2-BBU mixed vapor

An open 4 mL vial containing 20 mg of E-D cocrystal was placed in a sealed 20 mL vial containing 1 mL of each bromoalkane solution. E-D powders were exposed under saturated vapor pressure in the closed vessel at room temperature. Obvious color changes were observed over time. All bromoalkanes isomer mixtures are volume ratio, such as 1:1 (v/v), 1:99 (v/v), 99:1 (v/v). Before measurement, the powders were heated at 40 °C to remove the surface-physically adsorbed vapor.

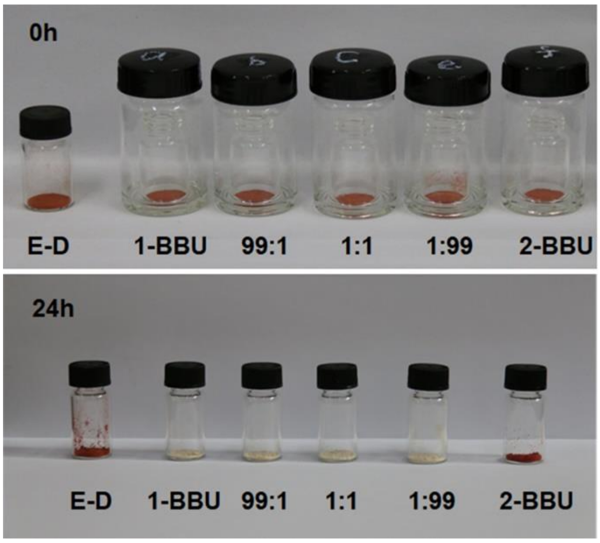

Figure S72. The color changes of vapor-phase adsorption experiments at 0 h and 24 h.

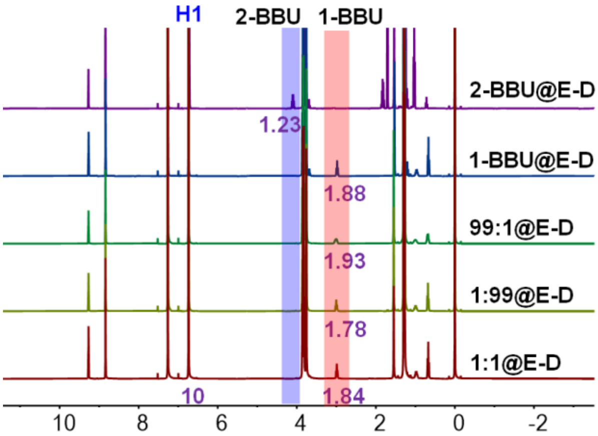

Figure S73. ^{1}H NMR spectrum (400 MHz, 298 K, $CDCl_3$) of 2-BBU@E-D, 1-BBU@E-D, 99:1@E-D, 1:99@E-D, 1:1@E-D (E-D cocrystal after adsorption in 1-BBU:2-BBU = 99:1 (v/v), 1-BBU:2-BBU = 1:99 (v/v), and 1-BBU:2-BBU = 1:1 (v/v) mixed vapor for 24 h). ^{1}H NMR experiments were performed by dissolving the E-D cocrystal powders after the vapor adsorption experiment in $CDCl_3$.

Table S3. Selectivity and adsorption capacity of 1-BBU.

Vapor-phase	1-BBU	2-BBU	1:99	1:1	99:1
Selectivity of 1-BBU	100%	-	97%	100%	100%
Adsorption capacity (n (EtP5: 1-BBU))	1:0.94	-	1:0.89	1:0.92	1:0.965
Adsorption capacity (n (EtP5: 2-BBU))	-	1:1.23	1:0.027	1:0	1:0

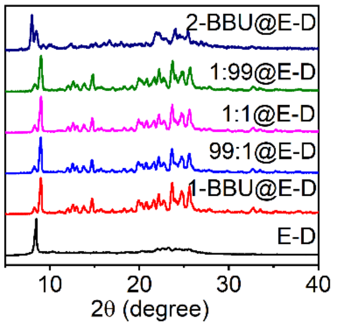

Figure S74. The PXRD patterns of E-D cocrystal, 1-BBU@E-D, 99:1@E-D, 1:1@E-D, 1:99@E-D, and 2-BBU@E-D.

9.3 Characterization of E-D cocrystal after adsorption 1-BPE:3-BPE mixed vapor

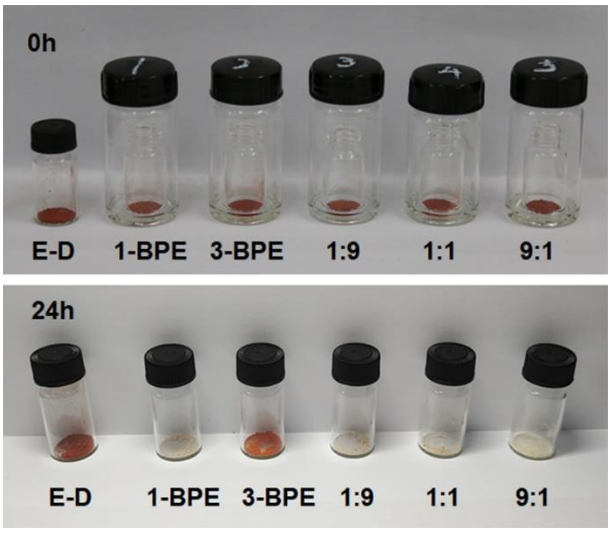

Figure S75. The color changes of vapor-phase adsorption experiments at 0 h and 24 h.

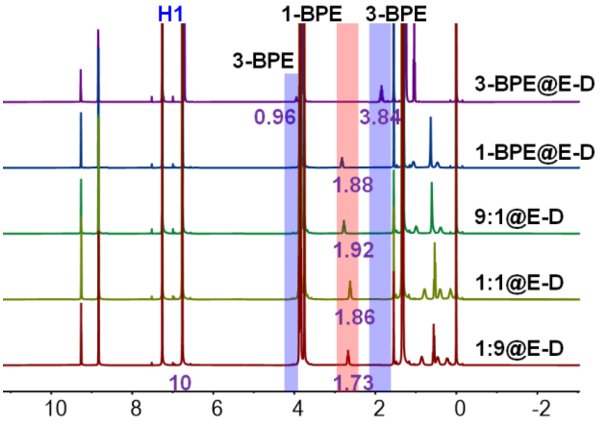

Figure S76. ^{1}H NMR spectrum (400 MHz, 298 K, $CDCl_3$) of 3-BPE@E-D, 1-BPE@E-D, 9:1@E-D, 1:1@E-D, 1:9@E-D (E-D cocrystal after adsorption in 1-BPE:3-BPE = 9:1 (v/v), 1-BPE:3-BPE = 1:1 (v/v), and 1-BPE:3-BPE = 1:9 (v/v) mixed vapor for 24 h). ^{1}H NMR experiments were performed by dissolving the E-D cocrystal powders after the vapor adsorption experiment in $CDCl_3$.

Table S4. Selectivity and adsorption capacity of 1-BPE.

Vapor-phase	1-BPE	3-BPE	1:9	1:1	9:1
Selectivity of 1-BPE	100%	-	100%	100%	100%
Adsorption capacity (n (EtP5: 1-BPE))	1:0.94	-	1:0.865	1:0.93	1:0.96
Adsorption capacity (n (EtP5: 3-BPE))	-	1:0.96	1:0	1:0	1:0

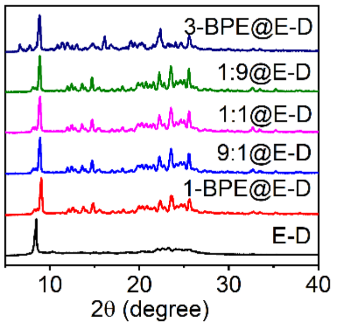

Figure S77. The PXRD patterns of E-D cocrystal, 1-BPE@E-D, 9:1@E-D, 1:1@E-D, 1:9@E-D, and 3-BPE@E-D.

9.4 Characterization of E-D cocrystal after adsorption 1-BHE:2-BHE mixed vapor

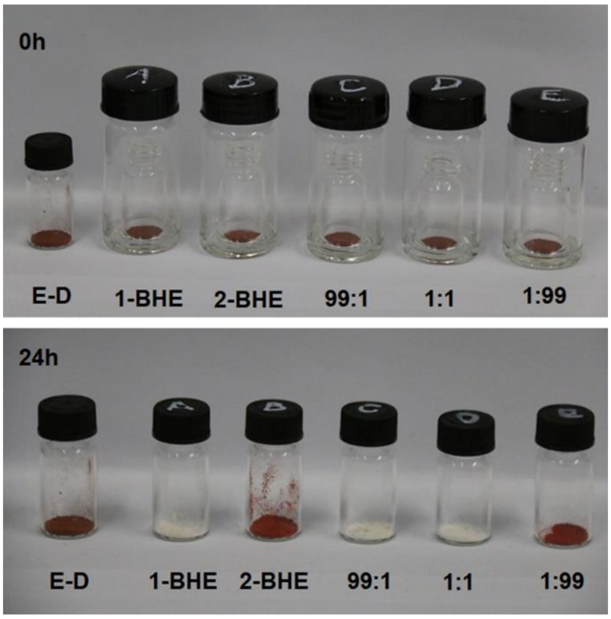

Figure S78. The color changes of vapor-phase adsorption experiments at 0 h and 24 h.

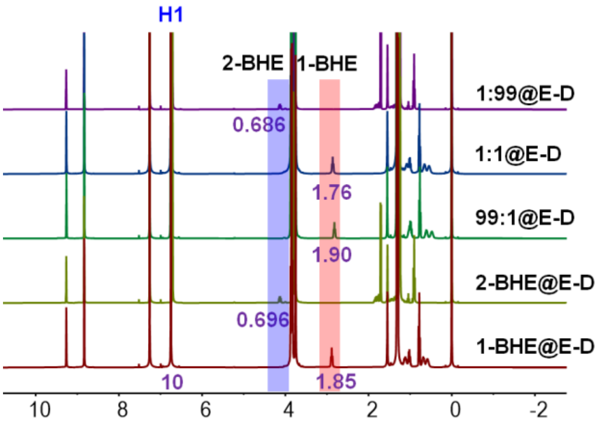

Figure S79. ^{1}H NMR spectrum (400 MHz, 298 K, $CDCl_3$) of 1-BHE@E-D, 2-BHE@E-D, 99:1@E-D, 1:1@E-D, 1:99@E-D (E-D cocrystal after adsorption in 1-BHE:2-BHE = 99:1 (v/v), 1-BHE:2-BHE = 1:1 (v/v), and 1-BHE:2-BHE = 1:99 (v/v) mixed vapor for 24 h). ^{1}H NMR experiments were performed by dissolving the E-D cocrystal powders after the vapor adsorption experiment in $CDCl_3$.

Table S5. Selectivity and adsorption capacity of 1-BHE.

Vapor-phase	1-BHE	2-BHE	1:99	1:1	99:1
Selectivity of 1-BHE	100%	-	8%	100%	100%
Adsorption capacity (n (EtP5: 1-BHE))	1:0.925	-	1:0.06	1:0.88	1:0.95
Adsorption capacity (n (EtP5: 2-BHE))	-	1:0.696	1:0.686	1:0	1:0
Adsorption capacity (n (EtP5: 3-BHE))	-	1:0.665	1:0.642	1:0	1:0

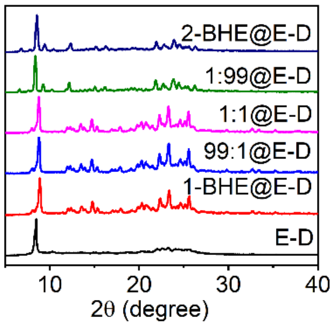

Figure S80. The PXRD patterns of E-D cocrystal, 1-BHE@E-D, 99:1@E-D, 1:1@E-D, 1:99@E-D, and 2-BHE@E-D.

9.5 E-D upon adsorption of the linear bromoalkanes exhibited excellent weathering resistance

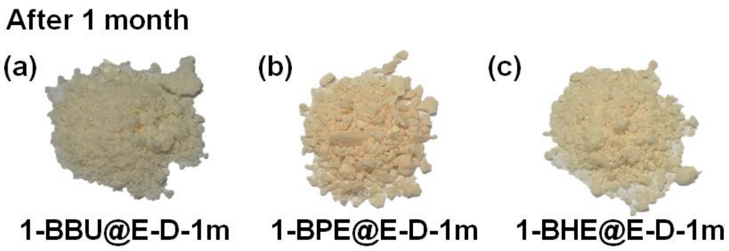

Figure S81. After 1 month, the color of linear bromoalkanes@E-D: (a) 1-BBU@E-D-1m, (b) 1-BPE@E-D-1m, and (c) 1-BHE@E-D-1m.

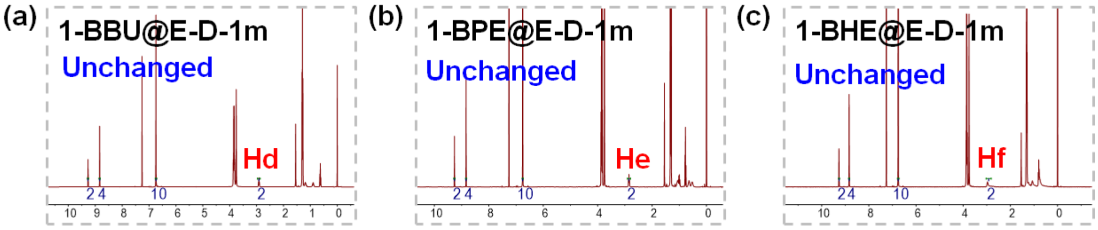

Figure S82. ^{1}H NMR spectrum of (a) 1-BBU@E-D-1m, (b) 1-BPE@E-D-1m, and (c) 1-BHE@E-D-1m.

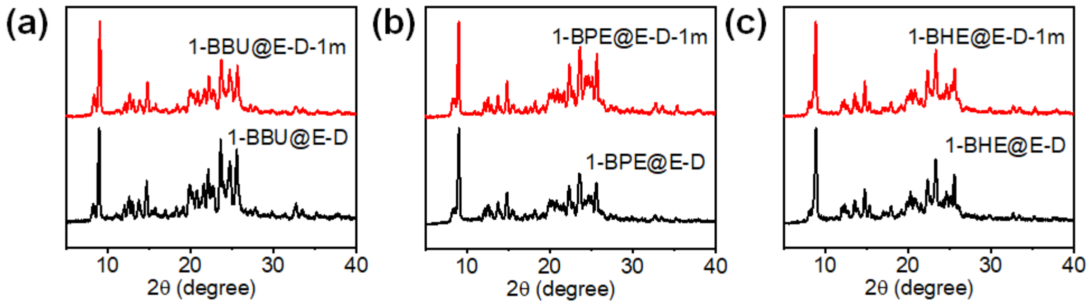

Figure S83. The PXRD patterns of (a) 1-BBU@E-D and 1-BBU@E-D-1m, (b) 1-BPE@E-D and 1-BPE@E-D-1m, and (c) 1-BHE@E-D and 1-BHE@E-D-1m.

10. Reversibility and recycling

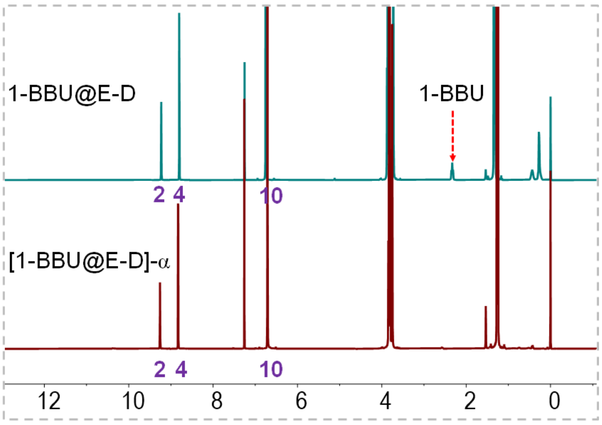

Figure S84. ^{1}H NMR spectrum (400 MHz, 298 K, $CDCl_3$) of 1-BBU@E-D and [1-BBU@E-D]-α (1-BBU@E-D desorption at 130 °C for 2 h).

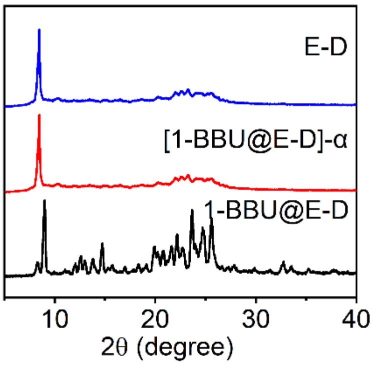

Figure S85. The PXRD patterns of 1-BBU@E-D, [1-BBU@E-D]-α, and E-D cocrystal.

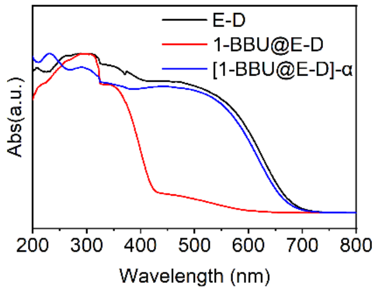

Figure S86. Normalized solid-state UV-Vis spectra of E-D cocrystal, 1-BBU@E-D, and [1-BBU@E-D]-α.

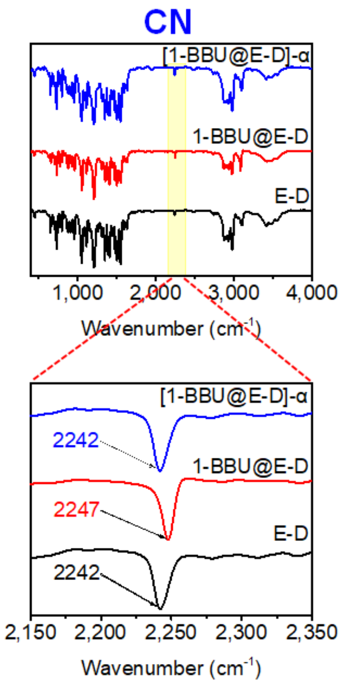

Figure S87. The FT-IR of E-D, 1-BBU@E-D, [1-BBU@E-D]-α, and the magnification of the CN group.

11. References

1. T. Ogoshi, S. Kanai, S. Fujinami, T.-a. Yamagishi, Y. Nakamoto, *J. Am. Chem. Soc.* **2008**, *130*, 5022-5023.
2. M. Wang, Q. Li, E. Li, J. Liu, J. Zhou, F. Huang, *Angew. Chem. Int. Ed.* **2021**, *60*, 8115-8120.
